# Magic angle spinning NMR structure of human cofilin-2 assembled on actin filaments reveals isoform-specific conformation and binding mode

**DOI:** 10.1038/s41467-022-29595-9

**Published:** 2022-04-19

**Authors:** Jodi Kraus, Ryan W. Russell, Elena Kudryashova, Chaoyi Xu, Nidhi Katyal, Juan R. Perilla, Dmitri S. Kudryashov, Tatyana Polenova

**Affiliations:** 1 Department of Chemistry and Biochemistry, University of Delaware, Newark, DE 19716 United States; 2 Department of Chemistry and Biochemistry, The Ohio State University, Columbus, OH 43210 United States; 3 Present Address: Department of Molecular Biology, Princeton University, Princeton, NJ 08544-1014 United States

**Keywords:** Solid-state NMR, Molecular modelling, Cytoskeletal proteins

## Abstract

Actin polymerization dynamics regulated by actin-binding proteins are essential for various cellular functions. The cofilin family of proteins are potent regulators of actin severing and filament disassembly. The structural basis for cofilin-isoform-specific severing activity is poorly understood as their high-resolution structures in complex with filamentous actin (F-actin) are lacking. Here, we present the atomic-resolution structure of the muscle-tissue-specific isoform, cofilin-2 (CFL2), assembled on ADP-F-actin, determined by magic-angle-spinning (MAS) NMR spectroscopy and data-guided molecular dynamics (MD) simulations. We observe an isoform-specific conformation for CFL2. This conformation is the result of a unique network of hydrogen bonding interactions within the α2 helix containing the non-conserved residue, Q26. Our results indicate F-site interactions that are specific between CFL2 and ADP-F-actin, revealing mechanistic insights into isoform-dependent F-actin disassembly.

## Abstract (web summary)

Despite their relevance as regulators of actin severing and filament disassembly, few structural insights into the mechanism of cofilin-isoform-specific severing activity are reported. Here, the authors provide structural insights towards actin severing activity by human cofilin-2 obtained by MAS NMR and all-atom MD simulations. The results reveal an isoform-specific binding mode unique to CFL2 that may be related to its potent severing properties in-vivo.

## Introduction

**Fig. 1:**
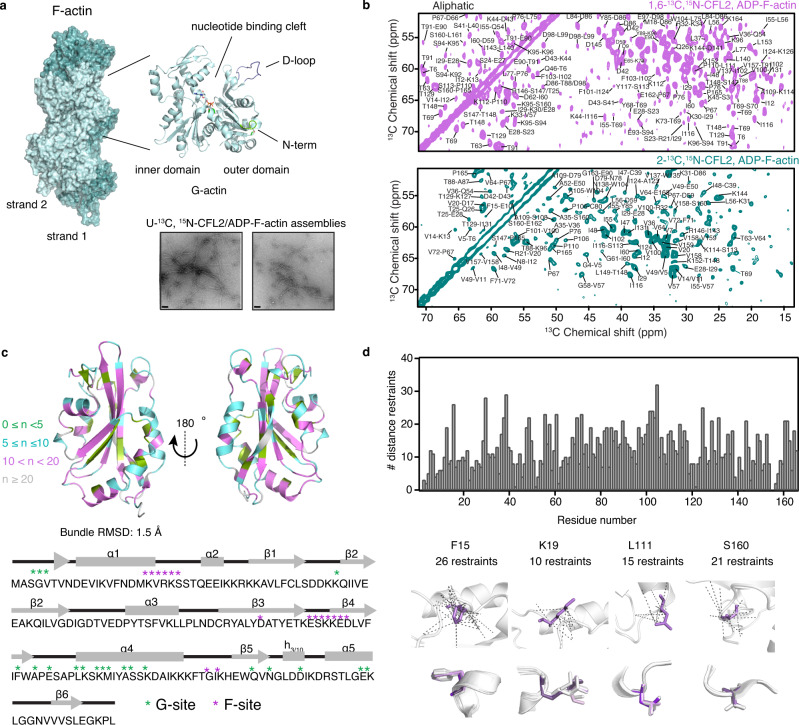
MAS NMR structure of CFL2 bound to ADP-F-actin. **a** Top: Structure of F-actin (left, PDB ID: 5ONV), composed of G-actin monomers (right, PDB ID: 1ATN). Separate strands are designated in pale cyan and dark teal. Bottom: TEM images of cofilactin assemblies used for MAS NMR experiments. A representative image before (left) and after (right) MAS NMR experiments is shown (scale bars: 100 nm). TEM images were collected on three independent preparations with similar results. **b** 2D ^13^C-^13^C CORD spectra acquired on CFL2 samples labeled with 1,6-^13^C-glucose (magenta) and 2-^13^C-glucose (teal) used for inter-residue distance restraints (CORD mixing time, 50 ms). Selected assignments are labeled on each spectrum. **c** Top: Ribbon representation of the lowest energy structure of CFL2 bound to ADP-F-actin (PDB ID: 7M0G). The number of unambiguous distance restraints are designated on the structure ranging from 0–5 restraints per residue (green) to greater than 20 restraints per residue (gray). Bottom: Sequence and secondary structure elements of CFL2. Residues constituting the canonical G- and F- binding patches are indicated with green and violet asterisks, respectively. **d** Top: Number of unambiguous distance restraints versus residue number. Bottom: Distance restraint networks and local alignment of structure ensemble for selected residues.

Monomeric (globular) actin (G-actin) undergoes dynamic cycles of polymerization into filamentous actin (F-actin) and depolymerization coupled to ATP hydrolysis, known as actin treadmilling (Fig. 1a)^1^. Actin treadmilling is the underlying mechanism for functions like cell migration and cell motility and is tightly regulated by different classes of actin-binding proteins (ABPs). These proteins work in concert to spatiotemporally carry out cellular functions^2^. The dynamics of F-actin is controlled by the actin nucleotide state, which acts as a so-called nucleotide clock. Nucleotide hydrolysis induces conformational changes at the intra-strand interface on the filament surface^3^, where they are sensed and amplified by several essential ABPs involved in the regulation of actin dynamics.

The cofilin family of proteins (referred to broadly as cofilin throughout the text) are responsible for actin filament severing and promote the turnover of G-actin monomers^4,5^. Cofilins are expressed in all eukaryotes, from yeast to humans. In humans, three separate genes encode for each cofilin isoform. Cofilin-1 (CFL1) is ubiquitously expressed; cofilin-2 (CFL2) is primarily expressed in muscle tissue; and destrin (DSTN, a.k.a. actin-depolymerizing factor) is expressed in neuronal and epithelial tissues. Cofilin is essential in mammals, and its different isoforms cannot fully compensate for each other in vivo due to their specialized roles^6^. For example, depletion of CFL1 in mice is embryonic lethal, and depletion of CFL2 causes abnormalities in α-skeletal muscle tissue development and fatal cardiomyopathies^7,8^. Each isoform also exhibits distinct depolymerization and severing rates, thus finely tuning actin dynamics in different cellular compartments to achieve the desired phenotype^9^. Consequently, aberrant cofilin–actin dynamics are associated with a range of diseases such as neurodegeneration^10^, multiple types of cancer^11^, HIV-1 infection^12^, and cardiomyopathy^13^.

Between human isoforms, there is >80% sequence homology, and the overall structure is highly conserved. Cofilin preferentially binds to aged F-actin containing ADP to promote filament severing and disassembly. While all cofilins have the highest affinity for ADP-F-actin, CFL2 can also interact with young ADP-Pi and ATP-enriched filaments through isoform-specific regions^14^. The canonical binding site on cofilin, determined by biochemical and biophysical techniques, consists of two patches of residues referred to as the G-site and the F-site^15–19^. Residues in the F-site can bind to bare actin filaments, albeit weakly, whereas F-actin must undergo conformational changes before G-site binding can occur^19^.

Cryogenic electron microscopy (cryo-EM) has been used to describe the changes in actin filament architecture that occur when cofilin is bound. However, the currently attainable resolution of 3.4 to 9 Å for different isoforms is insufficiently high for identifying fine structure details such as precise side chain orientations and hydrogen-bonding interactions. The structural basis for isoform-specific differences between DSTN, CFL1, and CFL2 is thus largely unknown because atomic-resolution structures of each isoform bound to F-actin have not been solved to date.

Here, we report the atomic-resolution structure of CFL2 uniformly decorated on ADP-F-actin, determined by magic-angle spinning (MAS) nuclear magnetic resonance (NMR) spectroscopy. The structure was calculated using 1277 non-redundant C-C distance restraints, extracted from correlation experiments, and 291 torsion angle restraints derived from ^13^C and ^15^N chemical shifts. An atomic-resolution structure of the CFL2/ADP-F-actin filament was determined by MAS NMR data-guided all-atom molecular dynamics (MD) simulations. The structure reveals a unique conformation of CFL2 that can form isoform-specific contacts with α-skeletal actin through an extended F-site binding surface thus providing structural insights into isoform-specific interactions between actin and its associated proteins.

## Results

### MAS NMR structure of CFL2 bound to ADP-F-actin

The assemblies of CFL2 with rabbit α-skeletal muscle actin (Fig. 1a) yield outstanding-resolution MAS NMR spectra, as shown in Fig. 1b and our previous work^20^. Overall, we recorded nine two-dimensional (2D) and two three-dimensional (3D) spectra on three sets of samples (summarized in Supplementary Table 1). On the basis of these data sets, we completed 96% of ^13^C and ^15^N chemical shift assignments using 2D ^13^C-^13^C combined R2-driven (CORD) and 3D ^15^N-^13^C-^13^C (NCACX/NCOCX) correlation spectra^21,22^. Chemical shift assignments are listed in Supplementary Table 2. Long-range ^13^C-^15^N and/or ^13^C-^13^C distance restraints were determined using [1,6-^13^C-glucose,U-^15^N]-CFL2/actin and [2-^13^C-glucose,U-^15^N]-CFL2/actin samples^23^, by recording proton-assisted insensitive nuclei cross-polarization (PAIN-CP) spectra^24^ and/or CORD spectra. In total, we assigned 1490 distance restraints corresponding to 1,224 unambiguous restraints and 238 ambiguous restraints (fewer than 4 possibilities per assigned cross peak), as summarized in Table 1. For this degree of restraint completeness, the accuracy of a structure should be within 0.8 Å backbone RMSD of the true structure^25^.

**Table 1:** Summary of MAS NMR distance constraints for CFL2 bound to ADP-F-actin.

Restraint category	^13^C-^13^C unambiguous (ambiguous)	^15^N-^13^C	Total
Total distance constraints	1224 (238)	25	1249 (238)
Intra-residue	580	2	
Sequential (|i-j| = 1)	244	13	
Medium range (1 < |i-j| <4)	162	5	
Long range (|i-j| >4)	238	5	
Torsion angle constraints			291

The structure of CFL2 assembled with actin is shown in Fig. 1c. Structure refinement and validation statistics are shown in Table 2. CFL2 exhibits an α/β domain fold typical of other cofilin isoforms^26–28^. The core consists of five β-strands surrounded by six helices and loop regions. β1–β4 form anti-parallel β-sheets, and β5 is packed parallel to β4. Compared to yeast cofilin, vertebrate cofilins are slightly larger and have sequence insertions in loop regions corresponding to the nuclear localization sequence (NLS) (residues R21-K33), residues L56-D66, and a short β-hairpin at the C-terminus (residues G155-L166). The bundle backbone and heavy-atom RMSDs for CFL2 are 1.05 and 1.67 Å, respectively. With the multiple distance restraints, the side chain conformations are well defined for 117 residues, with most sidechains adopting a single conformation (Fig. 1d).

**Table 2:** Summary of refinement and validation statistics for CFL2 structure (PDB ID: 7M0G).

Violations (mean ± s.d.)	
Distance constraints (Å)	0.055 ± 0.001
Dihedral angle constraints (Å)	1.198 ± 0.069
Max. dihedral angle violation (°)	8.0
Max. distance constraint violation (Å)	0.60
Deviations from ideal geometry	
Bond lengths (Å)	0.003 ± 0.000
Bond angles (°)	0.463 ± 0.009
Impropers (°)	0.37 ± 0.013
Average pairwise r.m.s.d. (Å)*	
Heavy	1.67 ± 0.12
Backbone	1.05 ± 0.15

^*^Pairwise r.m.s.d. was calculated among 25 refined structures for residues 6–165. Residues 1–5 and 166 are disordered.

### CFL2 exhibits an isoform-specific conformation distinct from other cofilin structures

**Fig. 2:**
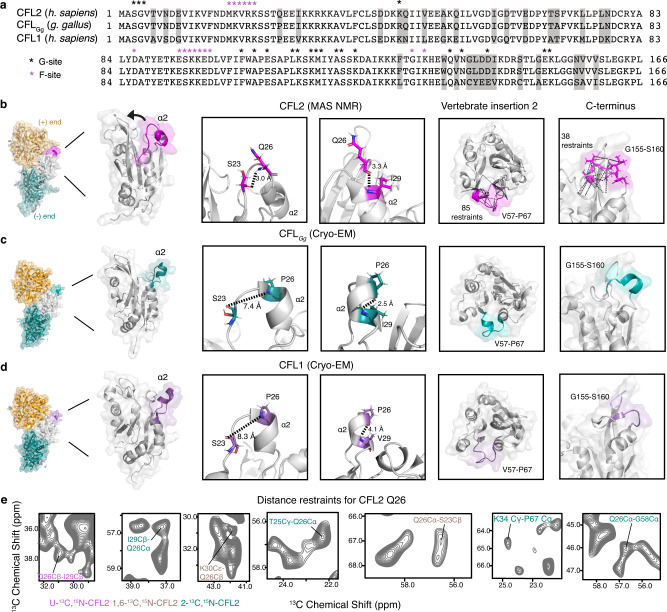
CFL2 exhibits conformational changes compared to other isoforms. **a** Sequence alignment of CFL2, CFL_Gg_, and CFL1. Non-conserved residues are indicated by gray shading. The canonical G-site (black) and F-site (magenta) binding sites are indicated by asterisks. **b**–**d** Left panels: Ribbon structures of CFL2 (pink, PDB ID: 7M0G), CFL_Gg_ (teal, PDB ID: 5YU8), and CFL1 (purple, PDB ID: 6VAO). Orientations of CFL isoforms bound between adjacent actin protomers (orange and teal) in the filament are shown. In each structure, the NLS containing α2 is indicated. CFL2 undergoes a 30° rotation of α2 which is not observed in other structures. Middle two panels: Expansions of α2 for CFL2 (magenta), CFL_Gg_ (teal), and CFL1 (purple). In CFL2, the P26Q substitution induces a remodeling of the hydrogen-bonding network in α2 that is responsible for the rotation. Right two panels: Regions corresponding to vertebrate insertion 2 and the C-terminal region for CFL2 (magenta), CFL_Gg_ (teal), and CFL1 (purple). The region containing vertebrate insertion 2 is structurally coupled to α2. For CFL2, this region is defined by 85 unique C-C restraints, represented on the structure with black dashed lines. The conformation of the C-terminal region of CFL2 is similar to CFL1 but exhibits more disorder compared to CFL_Gg_. For CFL2, this region is defined by 38 unique C-C restraints, represented on the structure with black dashed lines. **e** Unambiguous MAS NMR distance restraints containing Q26 and α2.

To elucidate isoform-specific features of CFL2, we compared our atomic-resolution structure to cofilactin structures containing *Gallus gallus* cofilin-2 (designated hereafter as CFL_Gg_) in a complex with chicken skeletal muscle F-actin (3.8 Å resolution, PDB ID: 5UY8) and human CFL1 in complex with mammalian α-skeletal F-actin (3.4 Å resolution, PDB ID: 6VAO)^19,29^. This comparison is shown in Fig. 2. Despite similarities in the protein core, we observe significant differences in surface regions of CFL2 containing the vertebrate-specific insertions (residues 18–34 and residues 57 – 67), as well as differences in other surface loops. These pronounced differences are surprising given that human CFL2 shares 98% sequence homology with CFL_Gg_. The most significant conformational change is a rotation of the α2 helix (α2) by approximately 30°. This change corresponds to a local RMSD of 4.04 Å between CFL2 and CFL_Gg_ for residues 21–32, while the global RMSD for the full-length proteins is only 1.84 Å (Supplementary Fig. 1 and Supplementary Table 3). This region is part of the vertebrate-specific insertion containing the nuclear localization signal (NLS). In human CFL2, α2 contains residue Q26, which is substituted by a conserved proline residue in other isoforms, including human DSTN and CFL1 as well as avian DSTN and CFL_Gg_. The substitution of proline with glutamine at residue 26 modifies the local structure around α2 and its preceding loop. Glutamine has a stronger helical propensity, and torsion angles derived from chemical shifts are consistent with a helix. This is also observed throughout the duration of the MD simulations on the entire cofilactin assembly, as discussed below.

The P26Q substitution induces remodeling of the local hydrogen bond network in α2 which reflects the differences in helical propensity between these two residues. Specifically, the Q26 backbone nitrogen atom forms a hydrogen bond with the backbone carbonyl atom of S23, and the mean hydrogen bond length is 3.1 Å. The Q26-S23 contacts are observed as cross peaks in the CORD spectra acquired with the mixing time of 500 ms (Fig. 2e), corroborating the presence of the interactions. In CFL_Gg_ and CFL1, where α2 is bent outwards, the distance between S23 and P26 is ~8 Å. Our CFL2 structure also reveals a hydrogen bond between Q26 and I29 (detected in the CORD spectra, Fig. 2e), whereas in other structures, P26 forms a hydrogen bond with K30. Interestingly, the NLS region of cofilin was proposed to act as a molecular switch^30^, which has to unfold in order to interact with cellular factors for nuclear import. Therefore, the different chemical properties of α2 and its preceding loop could facilitate this unfolding process with free energies for each isoform related to cell type-specific requirements.

Rotation of α2 also facilitates interaction between CFL2 residues S24 and E97 that has not been observed before^26,27^. In CFL_Gg_ structures where S24 and E97 are conserved, the distance from S24-Oγ to E97-Oε2 is ~13.0 Å. In our CFL2 structure, the distance between these residues is only 2.1 Å, well within hydrogen-bonding length. E97 is in β5, which is one major structural component of the F-site binding interface with F-actin. We speculate that this is a functionally important interaction, as outlined below.

The rotation of α2 induces allosteric changes to other mobile regions of CFL2, namely the loop containing residues G155-S160 and the loop containing the second vertebrate-specific insertion, V57-P67. The conformational change associated with the V57-P67 region is not surprising because it is structurally coupled to the first vertebrate-specific insertion (residues 18–34) which contains α2. Interactions between these two insertions (residues 18–34 and residues 57–67) were directly detected in the NMR spectra as cross peaks between the corresponding residues, as illustrated in Fig. 2e. We also observed structural differences in the loop containing residues G155-S160 near the C-terminus. Interestingly, the conformation of CFL2 in this region is more similar to CFL1 than to CFL_Gg_. In the structure of CFL_Gg_, this region is more structured and contains a short helix, whereas this region of CFL2 and CFL1 exhibits mostly loop content with a folded β-hairpin (Fig. 2e). This region is likely dynamic on NMR timescales, as G155 is one of the two residues (along with M1) not present in the spectra.

We have compared the atomic-resolution CFL2 MAS NMR structure determined here to the 9 Å resolution cryo-EM map of CFL2 assembled with ADP-F-actin^15^. Rigid body docking of the MAS NMR CFL2 structure to the cryo-EM density map is associated with a very high correlation score of 0.94 indicating that the main structural features of CFL2 are consistent in the structures from both techniques. The observed structural changes are localized to dynamic regions near the surface of the protein, containing several residues that interact with actin filaments. Taken together, our results suggest that this isoform-specific conformation of CFL2 is indicative of a unique binding mode on F-actin.

### Intermolecular interface reveals extended F-site on CFL2

Protein–protein interactions can be probed by MAS NMR spectroscopy through chemical shift perturbations (CSPs) that occur upon binding^31^ or by recording through-space correlations involving residues from each binding partner at the intermolecular interface, such as dREDOR experiments^32–34^. In our previous study, we used CSPs in combination with dREDOR-based experiments to investigate the intermolecular interface between CFL2 and ADP-F-actin. Since we obtained a complete set of resonance assignments in the present study, the results here offer unique insights compared to our earlier study^20^.

To determine the CFL2 residues forming the interface with ADP-F-actin, we used dREDOR-CORD, to measure correlations arising exclusively from the residues of CFL2 involved in binding to F-actin. The spectra reveal multiple correlations involving CFL2 residues at the N-terminus (A2-T6), the NLS (M18-Q26), R45, β4 (E90-E97), α4 (L111-K127), T129, K132, the 3_10_ helix (L140-D142), and α5 (E151-K152). While most of these regions are contained in the canonical interaction sites, to the best of our knowledge, this is the first report of the S24-Q26 region being part of the cofilin–actin binding site^20^. These data are summarized in Supplementary Fig. 2.

Since the structure of CFL_Gg_ was solved using solution NMR, we also performed an analysis of CSPs between CFL2 and CFL_Gg_ to validate the isoform-specific differences we observed between these structures. As expected, large CSPs were mapped to the three non-conserved residues (CFL2-Q26, R45, and S70), residues in the canonical G-site and F-site as well as to multiple residues in both vertebrate-specific insertions. These CSPs span residues at the interface with ADP-F-actin as well as residues that are far from the interface (likely due to allosteric effects). This is summarized in Supplementary Fig. 3.

### Atomic-resolution structure of cofilactin filament

The results of our MAS NMR experiments indicate specific interactions between CFL2 and F-actin that cannot be deduced from prior cryo-EM structures using other cofilin isoforms. To generate a complete atomic-resolution model of the entire CFL2/F-actin assembly (i.e., cofilactin), we employed data-driven all-atom molecular dynamics (MD) simulations using the distance restraint information from MAS NMR. The initial cofilactin model was prepared based on the 3.8 Å cryo-EM structure solved using CFL_Gg_ and F-actin^15,19^ (Supplementary Fig. 4), the isoform with the closest homology (98%), highest resolution structural model, and lowest structural variability between subunits. The cofilactin model maintains its structural integrity within 200-ns simulations. As shown in Supplementary Fig. 4b, c, root mean square displacements (RMSDs) of Cα atoms of the entire complex and the individual CFL2 chains plateau after about 25 ns. In addition, secondary structure assignments (STRIDE) of both actin and CFL2 subunits in the complex suggest that no global unfolding occurs, and the model is stable throughout the MD simulations.

To evaluate the protein–protein interactions in cofilactin, we calculated and compared the pairwise interatomic distances in the cryo-EM CFL_Gg_/F-actin structure, the initial cofilactin model built using our CFL2 structure, and the same cofilactin model at the end of 200-ns MD simulations (Supplementary Fig. 5). The results indicate that the cofilin–actin and the actin–actin interactions observed in the cryo-EM structure are conserved in our model and are sustained during MD simulations. Additionally, in our model, the Cα-Cα interatomic distances between CFL2 α2 (containing residues S24-Q26) and the actin N-terminus region are shorter compared to the cryo-EM model (Supplementary Fig. 5a, e, i), suggesting potential CFL2–actin interactions in this region not seen by cryo-EM.

**Fig. 3:**
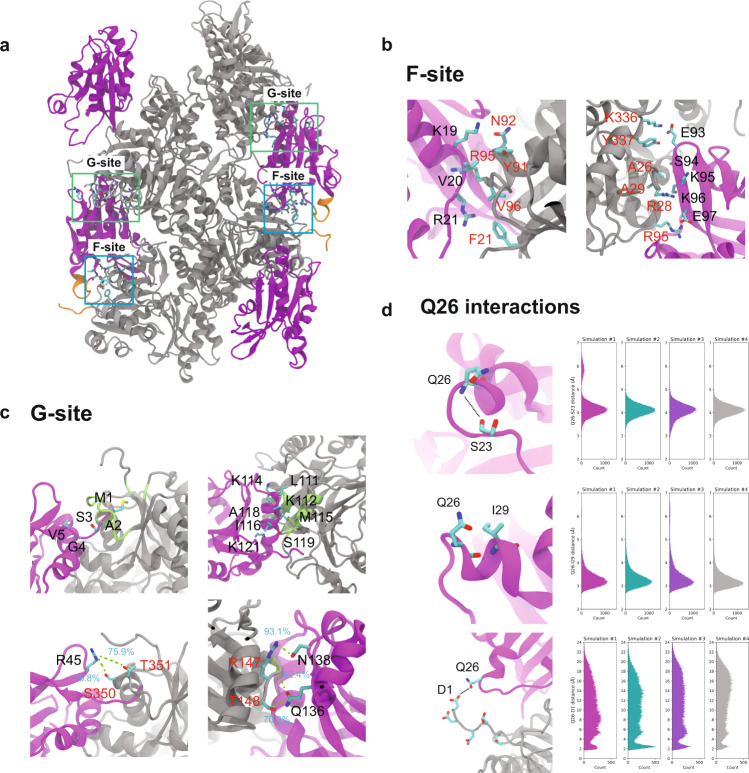
Cofilin-actin contacts observed during MD simulations. **a** The canonical binding sites of cofilin (magenta)/actin (gray) complex. The G- and F-sites are indicated with green and blue boxes, respectively. The region containing the N-terminus of actin and cofilin α2 are shown in orange. **b**, **c** CFL2 forms high-occupancy contacts with F-actin through the canonical F-site (**b**) and G-site (**c**) binding modes. **b** For the F-site on CFL2, these include the NLS, β4, and the loop preceding β5. **c** Residues located in the G-site of CFL2 include the N-terminus, α4, R45, and α5. For each binding interface, one representative CFL2 (magenta) and actin (gray) chain are shown. Cofilin and actin residues in the interaction interfaces are labeled in red and black, respectively. Contact occupancies of selected residues are shown on the structure. **d** CFL2-Q26 contacts observed during MD simulations. Cartoon representation of inter-residue contacts between Q26 and S23 (top left), I29 (middle left), and actin residue D1 (bottom left). One representative CFL2 (magenta) chain and one representative actin chain (gray) are shown for simplicity. Ensemble of the atomic distances for Q26-S23 (top right), Q26-I29 (middle right), and Q26–D1 (bottom right) obtained from MD simulations.

Contact analysis of MD trajectories identifies important CFL2–actin and actin–actin residue interactions. Fig 3 shows the CFL2–actin residue contacts with the highest contact occupancies >50% in seven regions, which fall into the canonical G- and F-site binding patches. These binding sites have been previously identified^15–19^ and correspond to cofilin residues that can bind directly to bare F-actin (F-site) as well as residues that require actin conformational changes prior to cofilin binding (G-site). In our model, residues in the G-site include CFL2 N-terminus interacting with actin residues 346–356, CFL2 α4 helix (residues 111 to 121) interacting with actin residues 140 to 149 and residues 341 to 349, CFL2 residue 45 interacting with actin residues 350 and 351, and CFL2 residues 129 and 132 interacting with actin residues 50, 56, 92, and 93. The F-site includes CFL2 residues 19 to 21 interacting with actin residues 91 to 96, CFL2 residues 93 to 97 interacting with actin residues 26 to 29 and 336 to 337, and CFL2 residues 147 and 148 interacting with actin residues 136 and 138. These interactions and their contact occupancies are summarized in Fig. 3 and Supplementary Table 4, respectively. Similarly, high-occupancy residue contacts in inter- and intra-strand actin–actin interaction interfaces are shown in Supplementary Fig. 6. We observed strong conservation of actin residues involved in both intra-stand interaction categories previously defined. These include inner domain (ID)—ID contacts between adjacent actin subunits as well as only a single outer domain (OD)—ID contact involving R62. Likewise, the inter-strand interactions evident in our structure are reduced compared to bare ADP-actin filaments, but consistent with previous cofilactin structures. Overall, both the actin–actin and CFL2–actin residue contacts identified in our MD simulations are similar to those reported in other cofilin–actin structures^15,19,29,35^ and are consistent with our MAS NMR results.

In our MAS NMR structure of CFL2, we observe a unique conformation of α2 helix containing the non-conserved residue Q26. Q26 participates in hydrogen-bonding interactions with S23 and I29. Through dREDOR experiments, we also identified CFL2 residue Q26 as part of the cofilin–actin interface. To investigate this further, we calculated the interatomic distances between Q26 and S23, I29, as well as D1 from actin’s N-terminus in the MD simulations. Gratifyingly, Q26 was found to strongly interact with S23 and I29. This is evidenced by the fact that most of the interatomic distances are shorter than 5.0 and 4.2 Å between the backbone nitrogen of Q26 and the carbonyl oxygen of S23 as well as the carbonyl oxygen of Q26 and the backbone nitrogen of I29, respectively (Fig. 3c). Interactions between CFL2-Q26 and actin-D1 were not stable throughout the simulations, although approximately 10% of the distributions have a distance shorter than 3.0 Å. It is worth noting that additional interactions between CFL2-Q26 with actin-E2, D3, and E4 were also observed during the simulations, but their contact occupancies were lower than those for the Q26–D1 interaction. The less frequent interactions between CFL2-Q26 and actin N-terminus are likely due to the inherent flexibility of the N-terminus of actin, as evidenced by the large root mean square fluctuations (RMSF) in this region (Supplementary Fig. 7). Therefore, it is possible that this is a low-population state under the sample conditions used here. Additionally, cross peaks corresponding to Q26 in the ^13^C-^13^C CORD spectra exhibit low peak intensities and are missing in our 3D NCACX dataset, which indicates some flexibility in this region of the protein. Indeed, the NLS region of CFL1 exhibits mobility based on ^15^N T_1_ and T_2_ relaxation rates from solution NMR^26^. This interaction may still be important under severing conditions (i.e., at junctions between bare and decorated filaments) or in vivo during interactions with other ABPs.

Recent cryo-EM studies of bare actin filaments in all nucleotide states have provided insights into the actin structure^3,36^. Specifically, the conformation of the DNase I-binding loop (D-loop) has been visualized, and its state is found to be coupled to the identity of the bound nucleotide. In the AMP-PNP- (a non-hydrolyzable ATP analog adenylyl-imidodiphosphate) bound state (mimicking the ATP-state), the D-loop exhibits an “open” conformation where it forms an extended hydrophobic network with F375 from the C-terminus of the adjacent actin protomer. Conversely, in the ADP-bound state, the D-loop assumes a “closed” conformation where this bridge with the C-terminus is broken, and the filament becomes more flexible.

Cofilin preferentially binds to aged ADP-actin filaments and induces disorder in the D-loop upon binding^36^. However, any specific conformations of the D-loop present in cofilactin are still unclear and have not been directly visualized in previous structures containing cofilin^19^. In our structure, the D-loop samples a conformation similar to the closed state, and there are no extended interactions with the C-terminus of the neighboring subunit. We also observed frequent interactions of the D-loop with CFL2 residues D122 and K126 in the long α4 helix, which are part of the interface. However, due to the flexibility of the D-loop, these interactions are not stable. To investigate this further, we calculated the conformational dynamics of the D-loop from MD trajectories and identified several free energy minimum states and metastable states (Supplementary Fig. 8). In most states, D-loop exists as a coil, but also forms turn, and even a helix in certain states (Supplementary Fig. 8). Interestingly, the helical conformation has only been observed experimentally in the X-ray crystal structure of ADP-bound G-actin^37^, and has not been seen in the recent cryo-EM structures. From our MD results, we observe that most of the barriers for conformational transitions between these states are smaller than 2 kT, confirming the flexibility of the D-loop, and its ability to easily transition between sub-states.

### In vitro severing activity of F-actin by CFL1 and CFL2

**Fig. 4:**
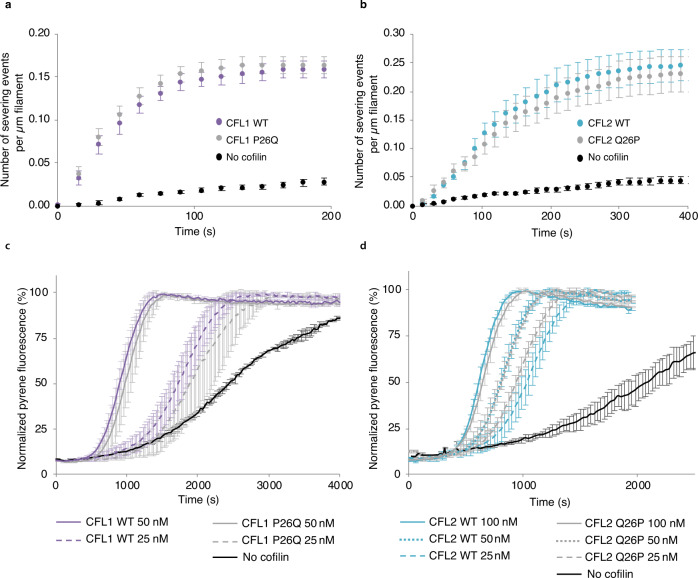
In vitro F-actin severing by CFL1 and CFL2 mutants. F-actin severing activity of cofilins was assessed through TIRFM analysis (**a**, **b**) and bulk pyrenyl-actin polymerization assays (**c**, **d**). **a**, **b** 0.9 μM Alexa488-actin (33% labeled) was polymerized in a TIRF chamber followed by the addition of human cofilins. Accumulation of severing events upon addition of (**a**) CFL1 WT (purple) and CFL1 P26Q (gray) or (**b**) CFL2 WT (blue) and CFL2 Q26P (gray) was counted and normalized per filament length prior to the addition of cofilin. Due to its notably higher severing potency, CFL2 was added at a 12-nM concentration as compared to 120-nM of CFL1 to obtain similar severing rates. Data were presented as the mean of three independent experiments with four fields of view analyzed within each experiment; error bars represent the standard error of the mean. **c**, **d** 2.45 μM pyrenyl-actin (5% labeled) was polymerized in the presence of 2.65 μM profilin-1 and indicated concentrations of cofilin constructs (designated by solid and dashed lines). CFL1 WT is shown in purple, CFL1 P26Q mutant is shown in gray (**c**). CFL2 WT is shown in blue and CFL2 Q26P mutant is shown in gray (**d**). Fluorescence intensities were normalized to the highest signal for each trace. Experiments were conducted in duplicates; error bars represent the standard deviation of the mean.

Since Q26 is located in the α2 helix and is not conserved in CFL1 or CFL_Gg_, we hypothesized that this residue is important in actin disassembly. To test this assertion, we substituted Q26 in CFL2 with proline found at this position in CFL1 and CFL_Gg_. In addition, we prepared a CFL1 P26Q mutant, where P26 is substituted with glutamine, unique to CFL2. We then compared the F-actin severing activities of the generated cofilin constructs using single-filament total internal reflection fluorescence microscopy (TIRFM) and bulk pyrenyl-actin polymerization assays. These data were summarized in Fig. 4.

As reported previously^9^, wild-type (WT) CFL2 showed much higher severing activity as compared to WT CFL1 in TIRFM experiments (Fig. 4a, b). In contrast, we observed only a marginal decrease in activity for CFL2 Q26P versus the WT. Similarly, we observed only a marginal enhancement in activity for CFL1 P26Q. In other words, the mutants behave similarly to their parent WT proteins. Therefore, the activity of each isoform is not determined by residue 26 alone. These results are consistent for both, single-filament TIRFM experiments (Fig. 4a, b) and bulk pyrenyl-actin polymerization assays (Fig. 4c, d).

Taken together, the results suggest that severing activity is modulated by cooperative interactions between multiple residues forming cofilin–actin interfaces, indicating a more complex regulation of severing activity. While at this time we do not have additional data to understand the structural basis for severing activity by different cofilin isoforms, we note that, for yeast cofilin, it is only the mutations in the F-site that are responsible for differences in severing activity^38^. Within the F-site, there are multiple non-conserved residues between CFL1, CFL2, and DSTN that could synergistically tune the severing activity. This will be investigated in future work. It is also possible that under cellular conditions, there is a complex interplay between the combined effects from multiple non-conserved cofilin residues at the binding interfaces with actin and interactions with other ABPs that participate in severing.

## Discussion

The atomic-resolution structure of CFL2 bound to ADP-F-actin determined herein revealed isoform-specific differences in the α2 helix: α2 is rotated 30° with respect to those of CFL1 and CFL_Gg_ isoforms^19,26,27,29^. The P26Q substitution specific to CFL2 remodels the local hydrogen-bonding network in this region of the protein through its increased helical propensity. Compared to P26, the polarity of the Q26 side chain is more optimal for interactions with the highly acidic N-terminus of α-skeletal F-actin, which would extend the F-site binding surface. We speculate that the combined effect from multiple non-conserved surface residues in the actin-binding site, potentially including Q26 in α2, contributes to isoform-specific differences observed in vitro and in vivo studies. This points to a severing mechanism that could be structurally distinct from that of other members of the cofilin family of proteins.

Various cofilin isoforms are often used to study actin filament disassembly since the early 1990s^5,39,40^. However, it was only in the last decade that differences between the mammalian isoforms have been investigated systematically^9,14^. Consistent with the results in this study, bulk severing assays showed that CFL2 is more efficient at severing both muscle actin and cytoplasmic actin compared to CFL1 and DSTN^9^. More recent microfluidics-based experiments confirmed these findings and demonstrated that faster decoration of F-actin by CFL2 than by other isoforms could explain more efficient severing^41^. Despite these interesting observations, the structural basis of isoform-specific severing activity of actin by cofilins is not yet understood. As demonstrated in our current study, resolving the interactive partners at atomic resolution is important because the rotation of α2 uniquely observed in CFL2 is a subtle feature that could not be seen in the 9 Å cryo-EM reconstruction. The conformational change of α2 most likely arises from the P26Q substitution as opposed to a conformational change induced by binding. This is supported by our MAS NMR data and by other structures of cofilactin containing CFL1, which exhibit very few differences with both the solution NMR and X-ray crystal structures of free CFL1^26,28,29^. Additionally, we note that the MAS NMR structure reported in this work was determined using a large number of restraints (11 restraints per residue) in the region containing α2 (residues 20–34).

We hypothesize that this isoform-specific conformation has a role in actin binding. This hypothesis is supported by our current and previous findings, where a stretch of residues 24–26 was detected in the dREDOR-CORD spectra, indicative of interactions with F-actin. Furthermore, in our data-driven all-atom MD simulations of cofilactin, we observed shorter overall distances between the α2 helix and N-terminus of actin as compared to other structures.

Many ABPs have interaction sites with the N-terminus of F-actin, which protrudes from the filament surface. Tropomyosin is one such protein, whose binding to F-actin excludes cofilin binding. The actin sequence is 93% conserved between human isoforms, and nearly all of the sequence variations are located in the N-terminus. Interestingly, regulation of cofilin activity by tropomyosins is isoform-dependent, where muscle tropomyosins inhibit CFL2 most efficiently^42^. This can be rationalized by our structure since tropomyosin could competitively bind with CFL2 at the N-terminus of F-actin through isoform-specific interactions. Moreover, mature actin is N-terminally acetylated^43^, which presents an additional degree of the regulation for actin binding and activity by ABPs.

Finally, it is of note that F-actin’s structural and functional polymorphism stems from intra- and intermolecular allosteric networks^3,36,44,45^. The flexible N-terminus of actin is coupled to the D-loop (subdomain 2) conformation, the hydrophobic plug, and the WH2-binding loop^44,46^. Interestingly, these regions of F-actin are involved in lateral and longitudinal contacts between subunits, and the D-loop is coupled to the nucleotide-binding cleft conformation^3,36^. We envision a mechanism where isoform-specific interactions and their interplay with actin nucleotide state, post-translational modifications, and competition with other ABPs drive distinct allosteric changes within the actin filament, leading to the phenotypes observed in cellular studies.

## Methods

### Materials

^15^NH_4_Cl, U-^13^C_6_ glucose, 2-^13^C-glucose, and 1,6-^13^C-glucose were purchased from Cambridge Isotope Laboratories, Inc (Tewksbury, MA, USA). Common chemicals were purchased from Fisher Scientific (Hampton, NH, USA) or Sigma Aldrich (St. Louis, MO, USA).

### Expression and purification of sparse-labeled CFL2

Cloning, expression, and purification of labeled tag-less human CFL2 was described previously^20^. Tag-less full-length human CFL2 was expressed in *Escherichia coli* BL21-CodonPlus(DE3) cells. Transformed cells were grown at 37 °C in 4 L of nutrient-rich medium supplemented with 50 mg/mL ampicillin and 34 mg/mL chloramphenicol until OD 1–1.2 was reached. Bacterial cells were pelleted, washed in MJ medium^47^ without glucose and ammonium chloride, resuspended in 0.75 L of MJ medium, and incubated for 1 h at 25 °C. U-^13^C_6_ glucose, 1,6-^13^C glucose, or 2-^13^C glucose was added to 4 g/L total concentration in addition to ^15^NH_4_Cl (1 g/L total concentration) and expression was induced using 1 mM isopropyl β-d-1-thiogalactopyranoside (IPTG). Cultures were grown overnight at 25 °C and pelleted at 4 °C the following day using buffer A [10 mM piperazine-*N*,*N*′-bis(2-ethanesulfonic acid) (PIPES), pH 6.8, 0.5 mM ethylenediaminetetraacetic acid (EDTA), 10 mM β-mercaptoethanol, 0.5 mM phenylmethylsulfonyl fluoride (PMSF), 5 mM benzamidine, protease inhibitor cocktail (Sigma Aldrich, St. Louis, MO, USA)] and lysed using a French cell press. Isotopically labeled CFL2 was purified using sequential anion and cation exchange chromatography followed by size-exclusion liquid chromatography, as described previously^20^.

### Preparation of F-actin

Skeletal muscle G-actin was prepared from acetone powder of rabbit skeletal muscle (Pel-Freeze Biologicals) according to the published method^48^ and stored in G-buffer (5 mM Tris-HCl, pH 8.0, 0.2 mM CaCl_2_, 0.2 mM ATP, 5 mM β-mercaptoethanol). G-actin was switched from Ca^2+^ to Mg^2+^ bound by incubating for 10 min with 0.1 mM MgCl_2_ and 0.4 mM EGTA. G-actin was polymerized as described previously^20^ by addition of buffer containing 20 mM PIPES pH 6.8, 25 mM KCl, 2 mM MgCl_2_, and 5 mM β-mercaptoethanol.

### Preparation of cofilin/actin assemblies

All cofilin-saturated F-actin assemblies were prepared using CFL2 and rabbit α-skeletal muscle actin at pH 6.6 to limit depolymerization, consistent with previous studies^15,20^. MAS NMR samples were prepared as follows. A solution of 2-^13^C,^15^N-CFL2, and F-actin were mixed at a 1:1.2 molar ratio and centrifuged at 435,400 × *g* at 4 °C for 1 h in a TLA 120.2 rotor using a Beckman Coulter Optima MAX-XP ultracentrifuge. A gel-like pellet was formed and centrifuged at 1700×*g* to transfer the pellet into a 1.9 mm Bruker rotor. 25.9 mg of CFL2/ADP-F-actin complex was packed into the 1.9 mm rotor, with an estimated 5.4 mg of isotopically labeled CFL2. The same procedure was employed for 1,6-^13^C,^15^N-CFL2/F-actin assemblies, except 16.8 mg of CFL2/F-actin complex was packed, which contained ~3.5 mg of isotopically labeled CFL2.

### Transmission electron microscopy

Sample morphologies for U-^13^C,^15^N-CFL2/ADP-F-actin, 2-^13^C,^15^N-CFL2/ADP-F-actin, and 1,6-^13^C,^15^N-CFL2/ADP-F-actin assemblies were confirmed using transmission electron microscopy (TEM). The assemblies were stained with uranyl acetate (5% w/v), deposited on 400 mesh formvar/carbon-coated copper grids, and dried. The TEM images were acquired using a Zeiss Libra 120 microscope operating at 120 kV. We acquired TEM images before and after MAS NMR experiments to confirm that no changes in filament properties occurred (Fig. 1a).

### MAS NMR spectroscopy

Spectra used for resonance assignments, distance restraints, and structure calculations were acquired at 19.96 T using a Bruker AVIII spectrometer equipped with a 1.9 mm HCN probe. Larmor frequencies were 850.4 MHz (^1^H), 213.8 MHz (^13^C), and 86.2 MHz (^15^N). All experiments were acquired at 14 kHz MAS, which was controlled to ±10 Hz by a Bruker MAS controller. The temperature was calibrated using KBr. The internal sample temperature was maintained at 273 K throughout the duration of data acquisition and controlled to ±0.1 °C with a Bruker temperature controller. ^13^C and ^15^N chemical shifts were referenced using adamantane (for ^13^C) and NH_4_Cl (for ^15^N).

The homo- and heteronuclear data sets used for resonance assignments are the same as those reported previously, and were discussed in detail^20^. Briefly, 2D and 3D NCACX and NCOCX experiments were obtained using nonuniform sampling (NUS). The 3D spectra were acquired with 25% NUS using 48 complex points in the t_1_ and t_2_ indirect dimensions, with maximum evolution times of 3.4 and 6.9 ms for ^13^C and ^15^N, respectively. The spectra were processed using the MINT reconstruction protocol. Typical 90° pulse lengths were 2.75, 2.95 μs for ^13^C, and 3.3 μs for ^15^N. The ^1^H-^13^C and ^1^H-^15^N CP employed a linear amplitude ramp of 80–100%, where the ^1^H RF field was 91 kHz. The center of the ramp on the ^13^C or ^15^N was Hartmann–Hahn matched to the first spinning sideband. In 2D and 3D NCACX experiments, the RF field strengths were 64.9, 84.7, and 91 kHz for ^15^N, ^13^C, and ^1^H channels, respectively. The DARR mixing sequence was applied to the ^1^H channel and the DARR mixing time was 50 ms. The ^1^H decoupling powers were 90–100 kHz during acquisition and evolution periods in all experiments.

For ^13^C-^13^C combined R2-driven (CORD) experiments^21^, typical pulse lengths were 2.6 μs for ^13^C and 2.8 μs for ^1^H. ^1^H-^13^C CP used a tangent amplitude ramp of 80 – 100%, where the ^1^H rf field was 75 kHz. The center of the ramp on ^13^C was Hartmann–Hahn matched to the first spinning sideband. The rf field was matched to the spinning frequency (14 kHz) and half of it (7 kHz) during 50, 200, and 500 ms CORD mixing times. Average ^1^H decoupling power was set to 90 kHz throughout acquisition and evolution. The ^13^C carrier frequency in both the direct and indirect dimensions was set to 95.0 ppm. ^13^C-^15^N PAIN-CP experiments^24^ were obtained on 2-^13^C,^15^N-CFL2/ADP-F-actin. The rf field strength of 60 kHz was used for ^1^H, ^13^C, and ^15^N during the PAIN-CP mixing time of 5 ms. Additional conditions (^1^H 90° pulse lengths and cross-polarization conditions) are the same as reported previously for NCACX and NCOCX spectra^20^.

MAS NMR data were processed with Bruker Topspin version 3.5 and NMRPipe version 8.7. NMR spectra were visualized and analyzed using CcpNmr Analysis version 2.4.2.

### Assignment of inter-residue distance restraints

^13^C-^13^C and ^13^C-^15^N inter-residue assignments were completed by manual analysis of a CORD spectrum acquired at 200 ms mixing time on uniformly-labeled CFL2, CORD spectra acquired at 50, 200, and 500 ms mixing time on 2-^13^C,^15^N-CFL2 and 1,6-^13^C,^15^N-CFL2, and PAIN-CP spectrum acquired on 2-^13^C,^15^N-CFL2. Many cross peaks in these spectra had multiple assignment possibilities based on chemical shift. Assignments were considered unambiguous using the following criteria: if one possibility corresponded to an intra-residue correlation, if one possibility corresponded to a sequential correlation, or if multiple cross peaks in the same spectrum were present and corresponded to the same spin system. If the correlation did not meet these criteria and could not be assigned to one unambiguous restraint based on the isotopic labeling scheme, we employed a distance filter approach to identify as many unambiguous assignments as possible. Specifically, assignment possibilities were filtered based on known distances from the solution NMR structure of chicken cofilin CFL_Gg_^27^, the most closely related isoform (PDB ID: 1TVJ). Any correlations that corresponded to >9 Å in the solution NMR structure were discarded as an assignment possibility. Due to the fact that our human CFL2 structure exhibited conformational changes in the α2 helix compared to chicken CFL_Gg_, another round of manual inspection of the distance restraint set was performed after our initial structure calculation. We manually inspected each restraint belonging to residues M18-K45 that were near the α2 helix. This ensured that no distance restraints were erroneously discarded or retained based on the structure of chicken cofilin.

### Structure calculation

Structure calculation was performed in XPLOR-NIH^49,50^ version 2.45. 1,277 unambiguous ^13^C-^13^C distance restraints, 335 ambiguous ^13^C-^13^C distance restraints, 25 ^13^C-^15^N distance restraints, and 291 TALOS-N^51^ torsion angle restraints were used as input in the structure calculation. Correlations from the CORD spectra were converted to a distance boundary of 1.5–6.5 Å for intra-residue correlations and 2.0–7.2 Å for inter-residue correlations^25^. The structure calculation of CFL2 was performed with similar input parameters used in our previous work^25^. In addition to potential energy terms that corresponded to distance restraints and torsion angle restraints, standard XPLOR-NIH energy terms were included. Standard terms for a bond, bond angle, and improper torsion angles were used to ensure proper covalent geometry. The gyration volume term was initialized excluding residues 1–5, and 166 to exclude disordered tails. A hydrogen bond database term, HBPot, was used to improve hydrogen bond geometries^52^. Backbone dihedral angle (ϕ, ψ) restraints were predicted using TALOS-N^53^ version 4.12 from experimental ^13^C and ^15^N chemical shifts.

An extended structure was generated based on the primary structure, and 250 structures were generated using torsion angle dynamics and simulated annealing followed by a final gradient minimization in Cartesian space using a Powell energy minimization scheme. The initial simulated annealing was started at 5000 K with a high-temperature run for 800 ps or 8000 steps, whichever finished first. Following the high-temperature run, the temperature was lowered to a final temperature of 20 K in steps of 20 K. At each temperature, a dynamics run was performed for 500 steps or 0.4 ps, whichever finished first. After this initial simulated annealing step, a refinement was performed using the 25 lowest energy structures. The refinement consisted of a second simulated annealing step from 3000 to 20 K in steps of 4 K. Force constants for the distance restraints were ramped from 10 to 50 kcal/mol/Å^2^ in the initial annealing step and from 2 to 30 kcal/mol/Å^2^ in the refinement step. We generated 250 structures during the refinement step and chose a representative bundle of the 25 lowest energy structures for further analysis. The coordinates corresponding to the atomic-resolution structures in this work have been deposited in the Protein Data Bank under accession code PDB 7M0G for CFL2. MAS NMR chemical shift, distance restraints, and dihedral angle restraints have been deposited in the Biological Magnetic Resonance Data Bank (BMRB) under accession code 30877. Structure calculation scripts can be provided upon request.

### Rigid body docking into cryo-electron microscopy density

Rigid body docking of CFL2 into the 9 Å resolution cryo-EM density map of cofilactin containing CFL2 and rabbit α-skeletal actin EMDB-5354 (PDB ID: 3J0S) was performed in UCSF Chimera version 1.13^54^. Rigid body docking only involved translation and rotation of CFL2 and did not alter side chain orientations. The rigid body docking procedure was a global search with 10,000 random orientations (5000 rotation orientations + 5,000 translations) to achieve the best fit between the MAS NMR atomic coordinates and the cryo-EM density map. The best fit was identified on the basis of highest cross-correlation scores; in this specific case the score was 0.94 on a scale of 0 – 1.

### Construction of atomic model for CFL2/F-actin

The initial coordinates of CFL2 were used directly from MAS NMR structure calculation. The initial structure of CFL2/F-actin was built using the cryo-EM structure of chicken cofilactin (PDB ID: 5YU8). The sequence of rabbit actin used in this study is identical to chicken actin, while CFL2 and CFL_Gg_ have a sequence conservation rate of 98.2%. The initial coordinates of actin were used directly from 5YU8. Missing actin residues in the N-terminus (residue 1 to 5) and D-loop (residue 41 to 49) were modeled in Modeller9.21^55^. Following this, the actin helical assembly was constructed using the experimental helical parameters^19^ (−162.1° twist and 27.6 Å rise). The Cα RMSD between the F-actin model built and the cryo-EM structure is 0.81 Å. Eight CFL2 molecules were fitted into the cryo-EM density (EMD-6844), and the model is shown in Supplementary Fig. 3a.

### Molecular dynamics simulations of CFL2/F-actin

Molecular dynamics simulations in this study were conducted using NAMD 2.14^56^ and CHARMM36m^57^ protein and CHARMM TIP3P^58^ water force fields were employed. After the CFL2–actin filament was constructed, NaCl ions were added to neutralize the filament and solvation of the whole system was performed using the TIP3P water^58^ model. Additional ions were added so that total bulk concentration of KCl was set to 25 mM and MgCl_2_ to 2 mM. The resulting model contains 563,000 atoms, including protein, ADP, the TIP3P water model, and ions.

The solvated system was then minimized for 10,000 steps using a conjugate gradient^59^ and line search algorithm^60^, with all backbone atoms of CFL2–actin filament fixed. The system was then heated from 50 to 310 K in 20 K increments for 1 ns while constraining the backbone atoms. Subsequently, the system was equilibrated for 10 ns. The equilibrated system was simulated in four independent NPT simulations and each one ran for 200 ns. In these simulations, the system temperature and pressure was maintained at 310 K at 1 atm using stochastic rescaling thermostat^61^ and Nosé–Hoover Langevin-piston pressure control, respectively. The backbone atoms in the helices of the first two and last two actin monomers were restrained with harmonic potentials (force constant of 0.5 kcal/mol-Å^2^) during simulations, to maintain the actin filament conformation. The eight cofilin molecules were applied with flat-bottom harmonic potential distance restraints derived from the experimental NMR data. All bonds to hydrogen were constrained with the SHAKE and SETTLE algorithm for the solute and solvent, respectively. Long-range electrostatic force calculations used the particle mesh Ewald method, with a 1.2 nm cutoff. The r-RESPA integrator and an integration time step of 2 fs were utilized, with the nonbonded interactions evaluated every 2 fs and electrostatics updated every 4 fs.

### Analysis of MD simulation trajectories

Contact, secondary structure, RMSD, RMSF, and pairwise distance and analysis were performed in VMD^62^. The contact is defined as the distance between sidechains of two residues are not greater than 3.4 Å. The contact occupancy was calculated by $\frac{{\sum }_{i=0}^{n-1}{\sum }_{j=0}^{m-1}{C}_{a,b}^{i,j}}{{\sum }_{i=0}^{n-1}{\sum }_{j=0}^{m-1}{C}_{{{{{{{\mathrm{total}}}}}}}}^{i,j}}\times 100 \%$, where *n* is the number of MD simulations, *m* is the number of interaction interfaces, ${C}_{a,b}^{i,j}$ is the number of frames in *i*th simulation and at *j*th interface where residue *a* and residue *b* form contact, ${C}_{{{{{{{\mathrm{total}}}}}}}}^{i,j}$ is the total number of frames in *i*th simulation and at *j*th interaction interface. The secondary structures of CFL2 and actin were assigned using the STRIDE algorithm. Homemade tcl scripts were written to compute the RMSDs, RMSF, and pairwise distances. Conformational dynamics of actin D-loop was calculated using time-lagged independent component analysis (TICA)^63,64^ implemented in pyEMMA 2.5.7^65^. Pairwise backbone atom distances in the D-loop (904 distance pairs) were chosen as the feature to partition the conformational space of D-loop. D-loop trajectories from four independent simulations were used as input data to run TICA. Note that there are 10 D-loops in each simulation but four of them in the actin molecules at two ends of the filament were not included in the TICA. TICA was computed with a selected lag time of 0.8 ns and the dimensionality of D-loop conformation was reduced to 10 independent components (ICs). The conformational dynamics of D-loop was projected onto the first two ICs.

### Structure analysis and visualization

RMS deviation values were calculated using algorithms in Xplor-NIH (version 2.51)^49,50^. Restraint tallying and format conversions were carried out with in-house Python 2.7 scripts. Structure ensembles were rendered for visualization in PyMOL 1.8.6.2 using in-house shell/bash scripts for batch rendering. Secondary structure elements were classified according to TALOS-N predictions and manual inspection. All analyses of cofilactin assemblies were performed in VMD.

### Cofilin mutagenesis and purification

For TIRFM and bulk pyrene-actin severing experiments, CFL1 and 2 were subcloned into a modified pColdI vector (Clontech) containing a 6xHis-tag followed by a TEV protease cleavage site^66,67^ using NEBuilder HiFi DNA Assembly Master Mix (New England Biolabs, Ipswich, MA, USA). Site-directed mutagenesis was carried out based on the Quick-change site-directed mutagenesis strategy (Agilent Technologies, Santa Clara, CA, USA) using Q5 DNA polymerase and DpnI restriction enzyme (New England Biolabs, Ipswich, MA, USA). The primer sequences used in this study are reported in Supplementary Table 5. WT and mutated cofilin constructs were expressed in *E. coli* BL21-CodonPlus(DE3) cells and purified using Talon metal affinity resin (Takara Bio USA, Inc., San Jose, CA, USA). 6xHis-tags were removed by treatment with recombinant TEV protease (1:20 w/w), which leaves a single glycine residue at the N-terminus. Following cleavage, 6xHis fragments, uncleaved 6xHis-constructs, and TEV protease were removed by passing through Talon metal affinity resin.

### TIRFM analysis of Alexa488-F-actin severing by cofilin

TIRFM experiments were conducted as described previously^68–70^ using the protocatechuic acid (PCA)/protocatechuate-3,4-dioxygenase (PCD) O_2_-scavenging system^71^. Skeletal actin (33% Alexa488-labeled, 1% biotinylated; 1.5 µM final concentration) was polymerized by the addition of an equal volume of 2× TIRF buffer in a TIRF flow chamber functionalized with 0.1 mg/ml streptavidin. Filaments were grown to ~15–20 µm average length. Free actin monomers were then removed by washing in cofilin in 1× TIRF buffer [final 1× buffer composition: 10 mM imidazole, pH 7.0, 50 mM KCl, 2.5 mM dithiothreitol (DTT), 1 mM MgCl_2_, 0.4 mM ATP, 0.2 mM ethylene glycol-bis(β-aminoethyl ether)-*N*,*N*,*N*,*N*-tetraacetic acid (EGTA), 10 mM ascorbic acid (neutralized to pH 7.0), 2.5 mM PCA (neutralized to pH 7.0), 0.1 µM PCD, 0.1% bovine serum albumin, and 0.5% methylcellulose-400cP (Sigma Aldrich, St. Louis, MO, USA)]. Due to its notably higher severing potency, CFL2 was added at a 12-nM concentration as compared to 120-nM of CFL1 to obtain similar severing rates (Fig. 4a, b). Time-lapse images were collected every 15 s using a Nikon Eclipse Ti-E microscope equipped with a TIRF module, perfect focus system, CFI Plan Apochromat λ ×100 oil objective (NA 1.45), and DS-Qi1Mc camera (Nikon Instruments Inc., Melville, NY, USA). Data were quantified using ImageJ software^72^: number of severing events was counted in each time frame and normalized to the filament length measured prior to the addition of cofilin. Data were presented as the mean of three independent experiments with four fields of view analyzed within each experiment.

### Bulk pyrenyl-actin polymerization assays

In a bulk mixture of 3.125 μM (5% pyrenyl-labeled) Ca^2+^-ATP G-actin with 3.44 μM human profilin-1 (PFN1; purified as described previously^69,73^), Ca^2+^ in the nucleotide cleft of actin was switched to Mg^2+^ by adding 0.02 volumes of 50× switch buffer: 500 mM 3-(*N*-morpholino)propanesulfonic acid (MOPS), pH 7.0, 5 mM EGTA, and 15 mM MgCl_2_. Forty-microliter samples were promptly transferred to a 96-well plate and supplemented with 0.1 of the final volume (5 μL) of cofilins present at concentrations tenfold higher than the desired final concentrations. Time-based monitoring of pyrene fluorescence in an Infinite M1000 Pro plate reader (Tecan US Inc, Morrisville, NC) was initiated with λ_ex_ = 365 nm and λ_em_ = 407 nm at 25 °C. In 2 min, using a multichannel pipette actin polymerization was initiated by adding 0.1 volumes (5 μL) of the 10× initiation buffer, containing 10 mM MgCl_2_ and 300 mM KCl. The samples were mixed promptly with a multichannel pipette set at 30 μL and the measurement was continued. The final concentrations of actin and PFN1 were 2.45 and 2.65 μM, respectively. Fluorescent intensity was normalized to the highest signal for each trace.

### Reporting summary

Further information on research design is available in the Nature Research Reporting Summary linked to this article.

## Supplementary information

Supplementary information

Reporting Summary

## Data Availability

The coordinates corresponding to the atomic-resolution structures in this work have been deposited in the Protein Data Bank under accession code PDB 7M0G for CFL2 and PDB 7U8K for cofilactin. MAS NMR chemical shift, distance restraints, and dihedral angle restraints have been deposited in the Biological Magnetic Resonance Data Bank (BMRB) under accession code 30877. The coordinates corresponding to actin filaments decorated with CFL_Gg_ used in this study are available in the Protein Data Bank under accession code PDB 5YU8. Additional coordinates corresponding to other proteins analyzed in this study are available in the Protein Data Bank under the following accession codes. The accession code for the solution NMR structure of CFL_Gg_ is PDB 1TVJ and corresponding BMRB entry 5177. The accession code for actin filaments decorated with CFL1 is PDB 6VAO. Source data for in vitro TIRFM severing and bulk pyrene-actin polymerization assays are provided within this paper. Other data that support the findings of this study, such as structure calculation scripts, are available from the corresponding authors upon reasonable request. Source data are provided in this paper.

## Source data

Source Data
