# Supplementary material for: Magic angle spinning NMR structure of human cofilin-2 assembled on actin filaments reveals isoform-specific conformation and binding mode
Source: Nat Commun. 2022 Apr 19;13:2114. doi: 10.1038/s41467-022-29595-9 (PMC9018683; doi:10.1038/s41467-022-29595-9)
Supplement: Supplementary file 1 — Supplementary information [file 41467_2022_29595_MOESM1_ESM.pdf]

Supplementary Information For

Magic Angle Spinning NMR Structure of Human Cofilin-2 Assembled on Actin Filaments Reveals Isoform-Specific Conformation and Binding Mode

Jodi Kraus[1,#], Ryan W. Russell,[1] Elena Kudryashova[2], Chaoyi Xu[1], Nidhi Katyal[1], Juan R. Perilla[1], Dmitri S. Kudryashov[2], Tatyana Polenova[1*]

[1]Department of Chemistry and Biochemistry, University of Delaware, Newark, DE 19716, United States; [2]Department of Chemistry and Biochemistry, The Ohio State University, Columbus, OH 43210, United States.

***Corresponding author:** Tatyana Polenova, Department of Chemistry and Biochemistry, University of Delaware, Newark, DE, 19716, United States, Phone: (302) 831-1968; Email: tpolenov@udel.edu

[#]Current address: Department of Molecular Biology, Princeton University, Princeton, NJ 08544-1014, United States

Supplementary Table 1. Summary of samples and MAS NMR spectra collected for resonance assignments and structure determination of CFL2 bound to ADP-F-actin.

Sample	Experiment	Mixing time (ms)
[U-^{13}C,^{15}N]-CFL2/actin	2D ^{13}C-^{13}C CORD	50<br>200
	3D ^{15}N-(^{13}CA)-(^{13}CX) with CORD mixing	50
	3D ^{15}N-(^{13}CO)-(^{13}CX) with CORD mixing	50
[2-^{13}C,^{15}N]-CFL2/actin	2D ^{13}C-^{13}C CORD	50<br>200<br>500
	2D PAIN-CP	5
[1,6-^{13}C,^{15}N]-CFL2/actin	2D ^{13}C-^{13}C CORD	50<br>200<br>500

Supplementary Table 2. ^{13}C and ^{15}N chemical shift assignments for CFL2 in complex with ADP-F-actin (BMRB accession code 30877).

Residue		^{15}N (ppm)	^{13}C' (ppm)	^{13}Cα (ppm)	^{13}Cβ (ppm)	^{13}C side chains (ppm)		
2	Ala	119.5	175.5	52.7	18.7			
3	Ser	109.7	174.6	58.2	63.9			
4	Gly	110	173.3	45	-			
5	Val	122.9	175.7	63.6	32.5	21.1:Cga	22.2:Cgb	
6	Thr	115.5	173.1	59.6	71.1	21.5:Cg2		
7	Val	118.4	174.9	61.2	32.3	21.6:Cga		
8	Asn	126.3	175.9	54.7	41.7	176.4:Cg		
9	Asp	124.8	177	57.8	40.4	179.3:Cg		
10	Glu	117.4	178.3	57.7	32.1	34.5:Cg		
11	Val	118.6	176.7	65.8	31	20.3:Cga	23.6:Cgb	
12	Ile	117	177.4	63.8	37.3	28.9:Cg1	17.9:Cg2	12.6:Cd1
13	Lys	120.5	178.4	59.8	32.2	24.8:Cg	29.1:Cd	41.8:Ce
14	Val	118.3	178.4	66	31.2	22.9:Cga	24.5:Cgb	
15	Phe	119.2	175.7	61.2	38.9	137.9:Cg	130.2:Cd*	131.0:Ce*
16	Asn	118.1	178	56.6	38.3	176.2:Cg		
17	Asp	119.3	178.3	56.1	40.3	179.3:Cg		
18	Met	119.9	178.4	58.7	33.6	18.4:Ce		
19	Lys	-	-	57	32.2	25.4:Cg	29.2:Cd	41.9:Ce
20	Val	114	174.3	62	32.4	20.8:Cga	22.0:Cgb	
21	Arg	129	176.6	56.9	29.7	27.6:Cg	43.2:Cd	159.2:Cz
22	Lys	123.2	177.8	56.6	32.4	25.5:Cg	29.1:Cd	42.0:Ce
23	Ser	121.3	178	57.8	67.3			
24	Ser	120.4	174.5	59.7	62.3			
25	Thr	112.9	175.6	61.8	69.5	22.1:Cg2		
26	Pro	113	176.7	56.7	30.9	32.6:Cg	180.2:Cd	
27	Glu	117	178.3	59.1	29.1	34.5:Cg	183.2:Cd	
28	Glu	118.2	180.2	58.4	29.3	36.8:Cg		
29	Ile	122.9	177.4	65.6	37.4	29.5:Cg1	16.8:Cg2	12.9:Cd1
30	Lys	114.2	178.3	58.9	32.5	25.3:Cg	29.0:Cd	41.8:Ce
31	Lys	115.7	175.8	55.7	32.4	25.7:Cg	28.8:Cd	
32	Arg	120.4	-	57	30.9	28.5:Cg	43.0:Cd	155.0:Cz
33	Lys	120.9	175.4	56.7	32.3	25.4:Cg	28.6:Cd	41.9:Ce
34	Lys	125.1	173.5	54.5	34.6	24.4:Cg	28.6:Cd	
35	Ala	115.7	175.2	50.9	23.3			
36	Val	116.2	171.7	60.4	35.2	21.5:Cga		

37	Leu	124.4	174.8	54.2	44.7	30.2:Cg	24.4:Cda	26.1:Cdb
38	Phe	116	174.3	55.6	41.5	139.7:Cg	132.8:Cd*	129.6:Ce* 127.5:Cz
39	Cys	116.7	172	53.9	32.8			
40	Leu	117.6	178.4	53.6	43.8	26.7:Cg	22.7:Cda	26.2:Cdb
41	Ser	117	176.5	58	63.9			
42	Asp	124.5	177.2	57.7	39.7	179.3:Cg		
43	Asp	114.9	175.5	52.9	39.5	179.6:Cg		
44	Lys	112.4	174.1	57.6	28.9	25.3:Cg	28.5:Cd	42.2:Ce
45	Arg	113.9	177	56.7	32.4	26.1:Cg		
46	Gln	114.2	173.9	53.3	34.3	179.3:Cd		
47	Ile	120.9	174	60.2	39.1	27.8:Cg1	19.0:Cg2	13.7:Cd1
48	Ile	119.4	175.3	59.7	42.3	26.2:Cg1	18.5:Cg2	14.0:Cd1
49	Val	119.4	175.5	63.3	32.5	21.3:Cga	22.9:Cgb	
50	Glu	131.1	175.8	54.7	28.7	36.8:Cg		
51	Glu	130.8	173.1	55.8	31.4	35.4:Cg	182.5:Cd	
52	Ala	127.2	176.3	51.3	19.8			
53	Lys	-	-	53	32.9	25.5:Cg	28.8:Cd	42.3:Ce
54	Gln	114.5	174.2	53.4	31.7	32.2:Cg	180.1:Cd	
55	Ile	118.5	174.8	59.4	40.7	27.1:Cg1	17.0:Cg2	12.9:Cd1
56	Leu	128.7	177.9	53	41.6	27.0:Cg	23.0:Cda	25.3:Cdb
57	Val	123.4	179.5	66.9	31.3	21.1:Cga	24.4:Cgb	
58	Gly	104.5	174	45.7	-			
59	Asp	117.7	178.3	56.1	41.1	179.3:Cg		
60	Ile	122	174.6	63.3	36.8	28.9:Cg1	17.1:Cg2	14.0:Cd1
61	Gly	111.2	175.2	42.5	-			
62	Asp	116.8	176.6	56.8	39.5	179.5:Cg		
63	Thr	112.5	175.3	60.6	70.5	21.7:Cg2		
64	Val	120.1	-	60.9	33.7	20.5:Cga		
65	Glu	119.1	178.3	59	29.1	35.8:Cg		
66	Asp	117.5	177.5	50	43.2	181.1:Cg		
67	Pro	135	175.9	64.4	31.7	26.8:Cg	50.8:Cd	
68	Tyr	118.5	176.7	63	38.2	131.5:Cg	131.9:Cd*	118.1:Ce*
69	Thr	114.1	177.7	66	68.4	21.9:Cg2		
70	Ala	115.3	174.9	62.1	63.3			
71	Phe	123.8	175.6	59.9	38	137.9:Cg	133.1:Cd*	129.1:Ce* 126.9:Cz
72	Val	118.9	178.1	65.9	31	21.7:Cga	23.1:Cgb	
73	Lys	114.4	177	57.7	32.2	25.4:Cg	30.1:Cd	41.8:Ce
74	Leu	117.2	177.4	55.4	42.8	25.2:Cg	22.2:Cda	25.1:Cdb

75	Leu	117.1	174.5	52.2	38	23.9:Cg	20.7:Cda	23.7:Cdb
76	Pro	135.8	177.4	61.6	31	26.6:Cg	50.0:Cd	
77	Leu	121.7	176.4	56.6	40.6	27.2:Cg	22.4:Cda	
78	Asn	110.7	174	50.6	38.4	111.4:Nd2	178.0:Cg	
79	Asp	113.6	172.6	52.9	43.2	180.9:Cg		
80	Cys	114.2	173.6	56.6	28.7			
81	Arg	116.4	-	53.9	38.2	26.8:Cg	43.1:Cd	159.0:Cz
82	Tyr	113.8	174.7	56.7	40.9	128.1:Cg	131.8:Cd*	118.0:Ce*
83	Ala	121.2	175.6	51.2	23.4			
84	Leu	118.1	173.8	53.4	46.7	27.4:Cg	24.7:Cda	26.6:Cdb
85	Tyr	122.6	172.7	56.8	42.3	131.9:Cg	131.8:Cd*	117.7:Ce*
86	Asp	128	172.9	52.1	40.1	181.4:Cg		
87	Ala	127.5	176.5	51.6	19.8			
88	Thr	122.2	173	61.7	69	22.1:Cg2		
89	Tyr	123.9	172	55.6	39	129.7:Cg	133.7:Cd*	118.0:Ce*
90	Glu	116.8	176.6	54.4	32.9	36.2:Cg	183.3:Cd	
91	Thr	114.3	174.7	58.8	71.4	22.0:Cg2		
92	Lys	116.2	176.8	58.5	32.5	25.7:Cg	29.2:Cd	41.8:Ce
93	Glu	112.2	176.2	56.6	32.8	36.3:Cg	184.2:Cd	
94	Ser	111.9	171.7	58.6	67.7			
95	Lys	123	175.4	56.7	32.4	25.4:Cg	29.1:Cd	42.7:Ce
96	Lys	125.1	174.9	54.7	34.7	24.2:Cg	27.9:Cd	42.1:Ce
97	Glu	118	176	55	32.3	36.5:Cg	184.1:Cd	
98	Asp	122.1	173.9	52	46.5	178.5:Cg		
99	Leu	118.6	176.5	55	43.7	26.8:Cg	23.6:Cda	
100	Val	121.6	174.6	61.4	34.6	20.5:Cga	21.1:Cgb	
101	Phe	126.7	173	56.4	40.8	138.8:Cg	132.0:Cd*	130.5:Ce*
102	Ile	129.1	173.3	60.2	42.1	29.9:Cg1	16.4:Cg2	15.3:Cd1
103	Phe	126.4	171.2	53.7	38.9	138.4:Cg	131.7:Cd*	130.1:Ce*
104	Trp	129.5	172.1	54.4	30.8	110.0:Cg	127.4:Cd1	129.1:Cd2<br>138.5:Ce2<br>119.8:Ce3
105	Ala	126.8	173.1	48.3	20.1			
106	Pro	137.5	176.4	61.3	31.1	26.8:Cg	48.4:Cd	
107	Glu	119.1	177.2	59	29.6	35.9:Cg	183.4:Cd	
108	Ser	107.9	174.3	58	62.6			
109	Ala	124.3	173.8	50.2	18.2			
110	Pro	133.3	177.2	62.2	32.1	27.9:Cg	50.5:Cd	
111	Leu	126.6	178.4	59	41.8	27.2:Cg	24.9:Cda	
112	Lys	114.7	178.1	60.5	32.1	26.0:Cg	29.3:Cd	41.8:Ce

113	Ser	110.7	175.4	61.7	63.2			
114	Lys	121.3	176.7	61.6	32.1	27.1:Cg	30.2:Cd	41.8:Ce
115	Met	113	178.4	58.8	32.4	30.6:Cg	18.1:Ce	
116	Ile	119.8	179	66.8	38.1	28.9:Cg1	18.4:Cg2	14.0:Cd1
117	Tyr	119.6	180	58.4	36.7	129.8:Cg	131.7:Cd*	117.9:Ce*
118	Ala	123.2	178.2	56.1	18.2			
119	Ser	108.8	174.8	60.9	63.1			
120	Ser	114	172.1	57.4	62.8			
121	Lys	121.4	178.4	58.7	-	24.0:Cg	41.9:Ce	
122	Asp	116.7	176	57	40.4	179.3:Cg		
123	Ala	118.8	179	54.4	17.7			
124	Ile	111.4	174.4	61.7	37.1	27.9:Cg1	17.2:Cg2	14.1:Cd1
125	Lys	121.9	178.8	58.6	31.7	24.0:Cg	29.2:Cd	41.8:Ce
126	Lys	114.2	177.8	57.8	32.1	25.4:Cg	29.1:Cd	41.9:Ce
127	Lys	114.1	176.2	54.6	31.4	24.2:Cg	27.9:Cd	42.6:Ce
128	Phe	122.2	174.5	51.2	34.9	138.5:Cg	129.0:Cd*	127.6:Ce*
129	Thr	112.9	175.8	61.8	69.5	21.9:Cg2		
130	Gly	111.9	175	45.2	-			
131	Ile	118.1	176.4	60.2	38.5	27.1:Cg1	18.4:Cg2	15.3:Cd1
132	Lys	125.7	175.5	57.1	33	24.7:Cg	29.2:Cd	41.9:Ce
133	His	116.6	174	55.6	32.7	129.9:Cg	125.5:Cd2	135.9:Ce1
134	Glu	124.1	174.5	54.5	31.7	36.2:Cg	182.3:Cd	
135	Trp	129.2	173.1	52.9	34	110.9:Cg	122.9:Cd1	136.8:Ce2<br>118.1:Ce3
136	Gln	130.9	172.9	55.9	31.4	35.4:Cg	181.5:Cd	
137	Val	126.8	173.9	61.2	34.1	18.7:Cga	23.5:Cgb	
138	Asn	122	173.5	53.1	40.7	107.1:Nd2	175.9:Cg	
139	Gly	103.7	173.4	45.5	-			
140	Leu	123.6	178.5	57.2	42.2	26.9:Cg	23.0:Cda	24.5:Cdb
141	Asp	116.8	-	56.6	40.5			
143	Ile	113.1	175.4	60.6	42.2	28.2:Cg1	15.8:Cg2	14.1:Cd1
144	Lys	115.3	174.7	54.7	34.7	24.3:Cg	28.6:Cd	42.1:Ce
145	Asp	115.9	177	53.5	42.4	180.0:Cg		
146	Arg	126.6	-	58.9	29.8	28.9:Cg	43.0:Cd	159.1:Cz
147	Ser	112.3	176.4	60.8	62.1			
148	Thr	117.7	177.5	65	67.8	22.6:Cg2		
149	Leu	125.5	178.3	56.6	38.5	26.8:Cg	22.2:Cda	26.9:Cdb
150	Gly	104	174.3	47.5	-			
151	Glu	119.4	178.5	59	29.1	35.8:Cg	183.2:Cd	

152	Lys	120.6	176.6	58.7	31.1	23.5:Cg	27.5:Cd	42.0:Ce
153	Leu	116.7	176.6	55.3	41.5	24.9:Cg	19.2:Cda	
154	Gly	103.1	-	47.7	-			
156	Asn	121.7	176.4	56.5	40.6	177.2:Cg		
157	Val	110.7	175.2	61.9	32.1	19.3:Cga	21.1:Cgb	
158	Val	121.4	175.3	63.6	31	22.3:Cga		
159	Val	121.1	176.4	61.6	33	19.2:Cga	21.0:Cgb	
160	Ser	115.1	171	56.9	64.6			
161	Leu	120.9	175.9	54.7	45.9	28.0:Cg	23.8:Cda	26.4:Cdb
162	Glu	126.6	176.4	55.5	25.8	34.2:Cg	182.9:Cd	
163	Gly	105.7	173.4	44.8	-			
164	Lys	120	178.4	52.3	33.6	24.6:Cg	28.9:Cd	42.0:Ce
165	Pro	136.2	176	62.8	32.1	27.1:Cg	50.9:Cd	
166	Leu	128.7	180.3	56.5	42.7	27.7:Cg		

Supplementary Table 3. Atomic RMSDs for the MAS NMR structure of CFL2 (PDB ID: 7M0G) compared to structural models of CFL1 and CFL_{Gg} from cryo-EM and solution NMR spectroscopy.

Isoform	PDB ID	Backbone RMSD (A)	Heavy atom RMSD (A)	Residues 21-32 backbone RMSD (A)	Residues 21-32 heavy atom RMSD (A)
CFL1 (cryo-EM)	6VAO	1.540	1.899	3.229	4.482
CFL1 (solution NMR)	1Q8G	2.139	2.375	2.732	3.813
CFL_{Gg} (cryo-EM)	5YU8	1.470	1.842	3.033	4.036
CFL_{Gg} (solution NMR)	1TVJ	1.495	1.772	2.794	3.642

Supplementary Table 4. Contact occupancies for CFL2-actin interfaces throughout MD simulations.

Binding site	CFL2 residue	Actin residue	Occupancy (%)
G-site	M1	W356	91.6
	M1	Q353	77.8
	M1	P102	69.4
	M1	S350	64.4
	M1	A347	59.7
	M1	T351	59.7
	M1	F352	53.6
	M1	A7	52.2
	A2	W356	75.8
	A2	L346	75.4
	A2	L349	60.7
	A2	M355	54.5
	S3	Q354	66.2
	S3	T351	63.7
	S3	F352	63.5
	S3	M355	62.3
	G4	L349	69.3
	V5	L349	73.5
	V5	T351	66.6
	L111	I341	97.9
	L111	A144	79.4
	K112	I345	100.0
	K114	G346	100.0
	K114	S145	82.3
	M115	Y143	100.0
	M115	I345	100.0
	M115	G342	98.7
	M115	A144	98.1
	M115	L346	97.0
	M115	L140	90.6
	I116	L349	98.5
	I116	I345	80.5
	A118	Y143	100.0
	A118	T148	95.5
	A118	G146	88.7
	S119	Y143	98.0
	K121	T148	100.0
	K121	E167	87.9
F-site	K19	R95	77.4
	K19	N92	55.1
	K19	Y91	51.6
	V20	R95	99.4
	V20	Y91	70.1
	V20	V96	55.6
	R21	R95	94.8
	R21	V96	89.3
	R21	F21	52.4
	E93	K336	93.0
	E93	Y337	87.8
	S94	A26	88.6
	S94	R28	82.0
	K95	R28	85.1
	K96	A29	78.6
	K96	R28	66.0
	E97	R95	96.9

Supplementary Table 5. Primers used in this study.

Primer	DNA Sequence	Usage
Forward	5’-TCTTTATTTCCAAGGTACCGCCTCCGGTGTGGCTGTC-3’	CFL1 subcloning into a modified pColdI vector
Reverse	5’-TAAGCAGAGATTACCTATCTAGATCACAAAGGCTTGCCCTCCAG-3’	
Forward	5’-TCTTTATTTCCAAGGTACCGCTTCTGGAGTTACAGTGAATG-3’	CFL2 subcloning into a modified pColdI vector
Reverse	5’-TAAGCAGAGATTACCTATCTAGATTATAATGGTTTTCCTTCAAGTGAAAC-3’	
Forward	5’-GTAAGTCTTCAACGCAAGAGGAGGTGAAGAAG-3’	CFL1 mutagenesis (P26Q)
Reverse	5’-CTTCTTCACCTCCTCTTGCGTTGAAGACTTAC-3’	
Forward	5’-GAAATCTTCTACACCCGAGGAGATCAAAAAG-3’	CFL2 mutagenesis (Q26P)
Reverse	5’-CTTTTTGATCTCCTCGGGTGTAGAAGATTTC-3’	

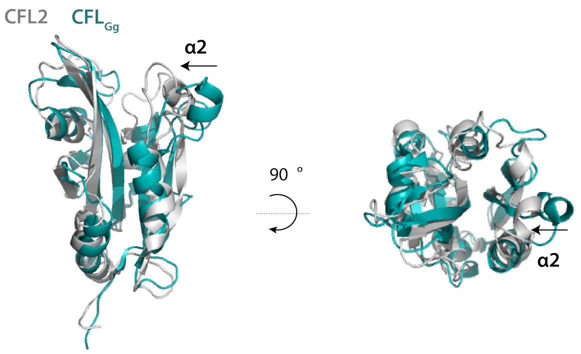

Supplementary Figure 1. Overlay of MAS NMR structure of CFL2 (PDB ID: 7M0G) and chicken cofilin-2 (PDB ID: 1TVJ).

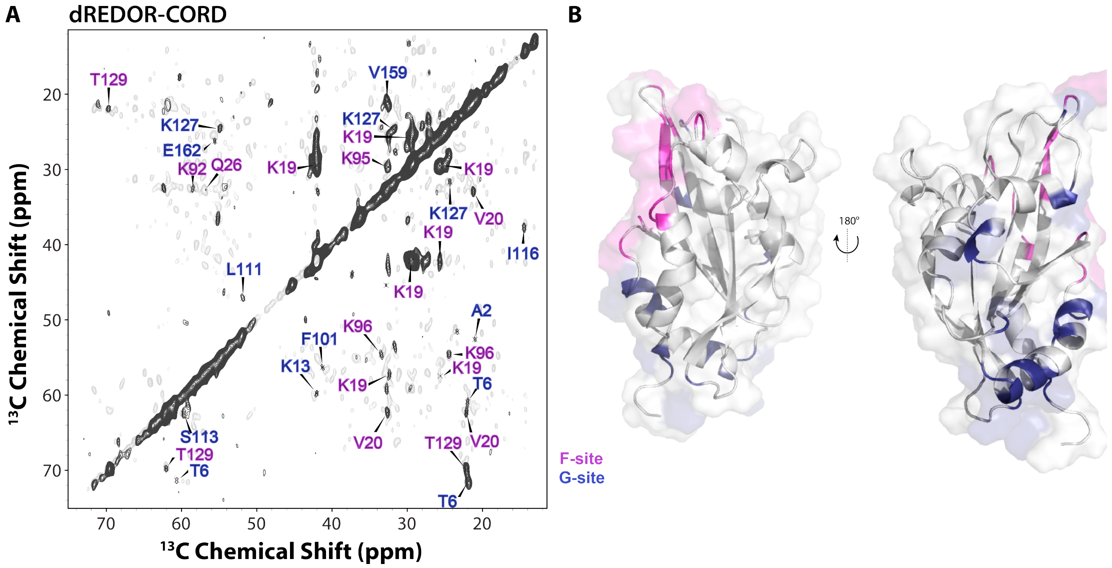

Supplementary Figure 2. CFL2 residues comprising the cofilin-actin interface as determined by dREDOR-CORD experiments. **A.** Overlay of dREDOR-CORD spectrum (dark gray) with 50 ms mixing time CORD spectrum (light gray) for U-^{13}C,^{15}N-CFL2/ADP-F-actin. Peaks shown in dark gray belong to residues at the CFL2/actin interface. Selected assignments are shown in blue (G-site) and magenta (F-site). **B.** Interface residues from dREDOR-CORD experiment mapped on the MAS NMR structure of CFL2.

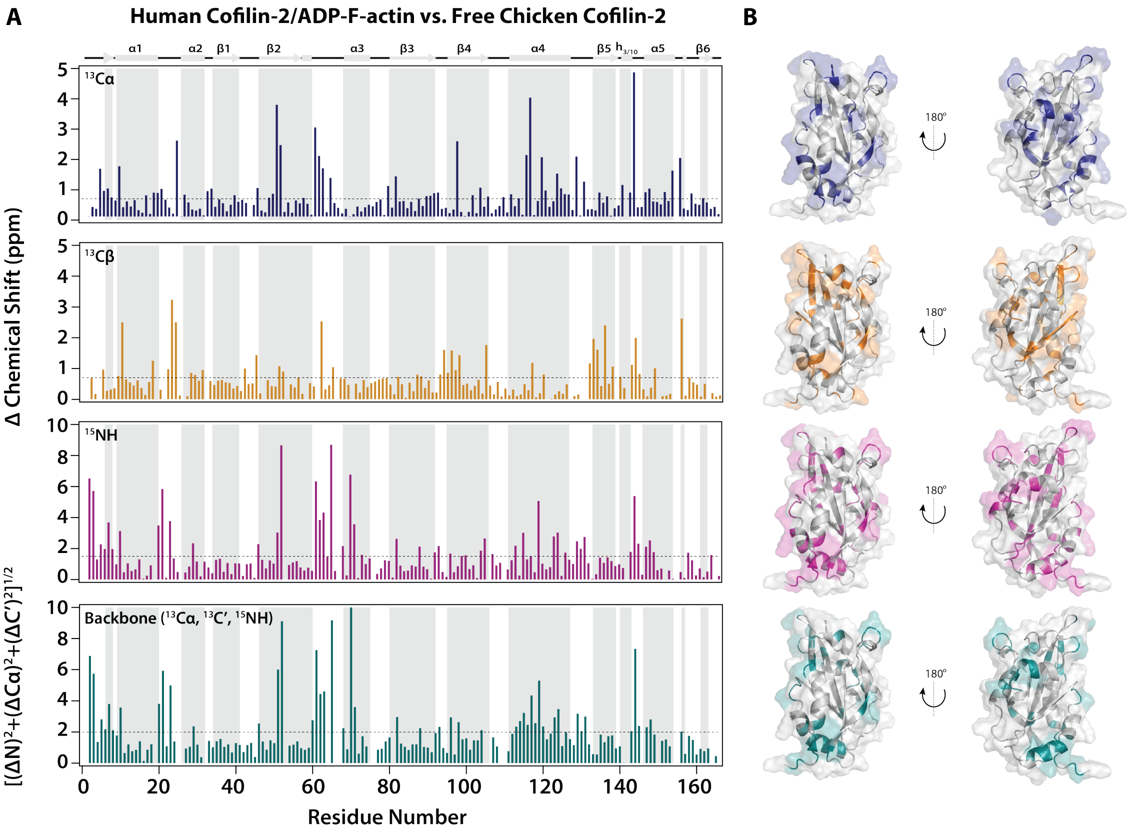

Supplementary Figure 3. Summary of chemical shift perturbations (CSPs) between MAS NMR chemical shifts of CFL2 in complex with ADP-F-actin (PDB ID: 7M0G, BMRB entry: 30877) and solution NMR chemical shifts of free CFL*Gg* (PDB ID: 1TVJ, BMRB entry: 5177). **A.** Absolute chemical shift differences as a function of residue number for ^{13}Cα (violet), ^{13}Cβ (orange), ^{15}N^{H} (pink), and weighted CSPs (teal). Any residue that has a chemical shift perturbation greater than the dashed line indicates a significant perturbation. **B.** Significant perturbations mapped onto the ribbon structure for the MAS NMR structure of CFL2.

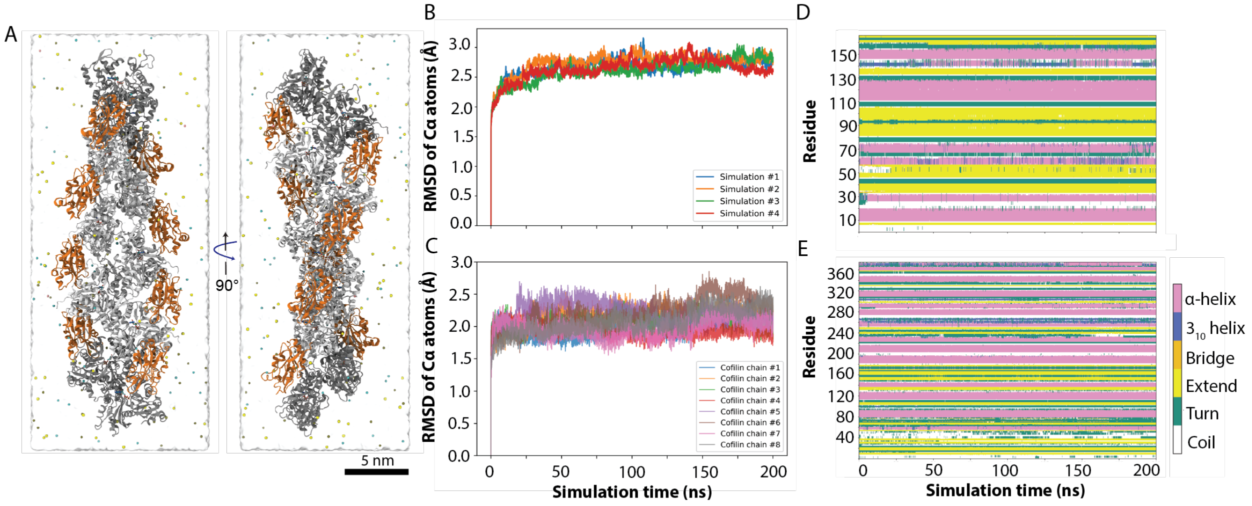

Supplementary Figure 4. Structural stability of CFL2-actin filament. (a) Model of CFL2-actin filament system used during molecular dynamics simulations. Secondary structure assignments of CFL2 (orange) and actin (gray) are shown. The filament contains 10 actin subunits decorated with 8 CFL2 molecules. The pointed end is located at the top of the panel. Each actin binds an ADP molecule. The ions in the system are represented as spheres, where different ions are differentiated by color, namely Mg ions in pink, sodium ions in yellow and chloride ions in cyan. The system was solvated with TIP3P water, which is illustrated as the transparent box surrounding the protein. (b) Root mean square displacements (RMSD) of Cα atoms in the CFL2-actin filament from four independent MD simulations. (c) RMSD of Cα atoms in eight CFL2 subunits from MD simulation #1. (d) and (e) Secondary structure assignments (STRIDE) of a CFL2 and an actin subunit in simulation #1. Simulations #2, 3, and 4 showed similar results (data not shown). The colors shown in the color bar in the lower left corner indicate different secondary structure assignments in panels d and e.

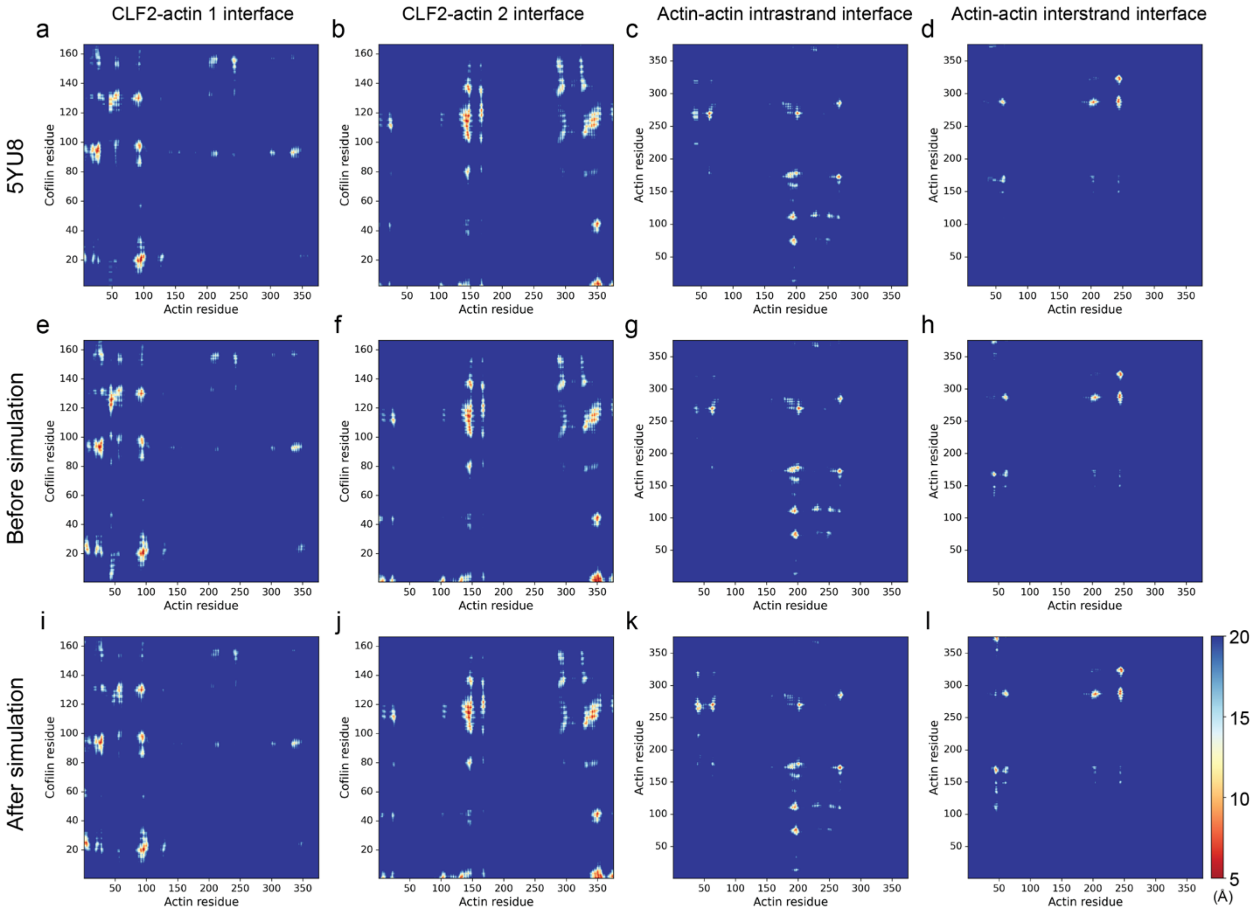

Supplementary Figure 5. CFL2-actin and actin-actin interactions, in cryo-EM structure (PDB: 5YU8) (a-d), cofilin-actin filament system built in the present study (e-h) and observed at the end of MD simulation (i-l). These interactions are obtained as pairwise Cα atom distances in CLF2-actin interfaces (a, b, e, f, i and j), actin-actin intra-strand interface (c, g and k) and actin-actin inter-strand interface (d, h and l). Pairwise distances are shown in the color bar. The “actin 1” interface refers to interactions in the F-site and the “actin 2” interface refers to interactions in the G-site.

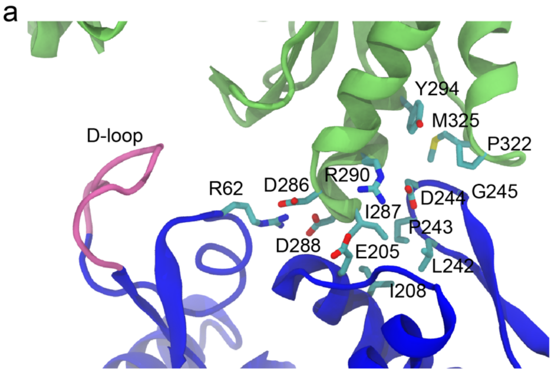

b

AC residues	AC residues	Occupancies(%)
**D244**	**R290**	99.8
**P243**	**I287**	99.0
**R62**	**D288**	90.6
**D244**	**Y294**	90.4
**I208**	**I287**	88.9
**G205**	**I287**	88.4
**G245**	P322	85.0
**L242**	**I287**	82.9
**Q246**	P322	82.3
**D244**	**I287**	79.9
**G245**	M325	78.4
**D244**	M325	78.1
**R62**	**D286**	74.4
**A204**	**I287**	74.4
**D244**	**K291**	71.4
**R62**	**I289**	65.4
**G245**	T324	64.6
**P243**	**K291**	61.9
**A204**	**D288**	60.1
**P243**	**D288**	56.6

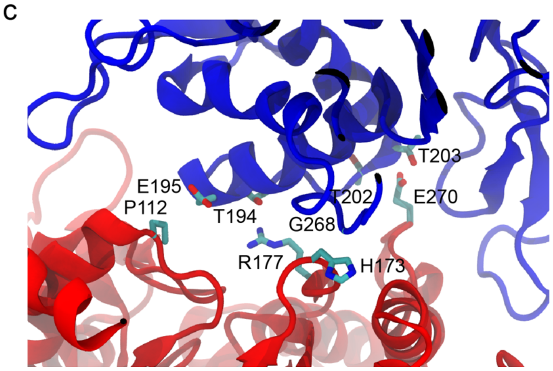

d

AC residues	AC residues	Occupancies(%)
**E195**	**P112**	95.3
**T202**	**E270**	93.6
**G268**	**H173**	85.7
**T203**	**E270**	85.0
**T194**	R177	80.6
**G63**	**E270**	69.1
**E195**	I75	65.5
**G197**	H73	55.9
**V201**	**S271**	55.7
**V201**	D179	55.0

Supplementary Figure 6. Actin-actin contacts observed during MD simulations. Actin-actin intra-strand and inter-strand interactions (a, c) and the contact occupancies in these interfaces (b, d). Previously reported residues that form actin-actin interactions are shown in a bold typeface.

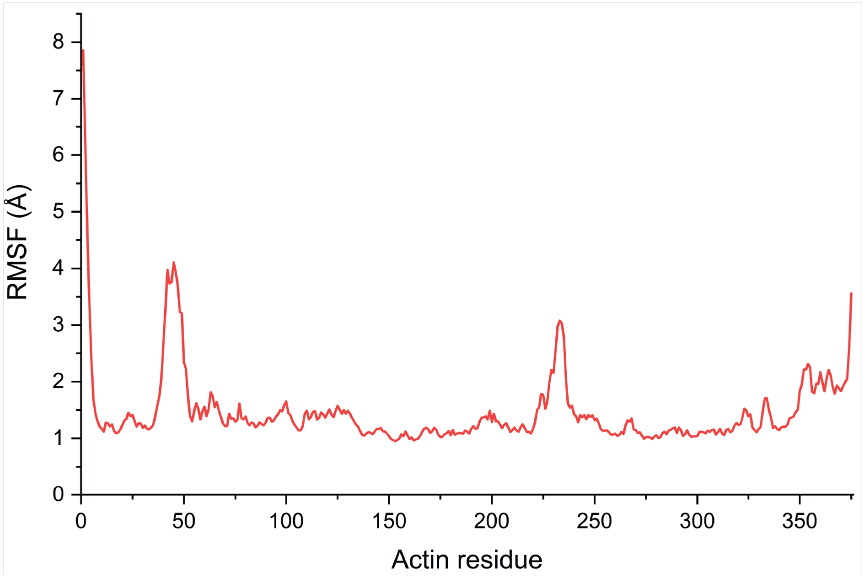

Supplementary Figure 7. Root mean square fluctuation (RMSF) of actin residues throughout MD simulation.

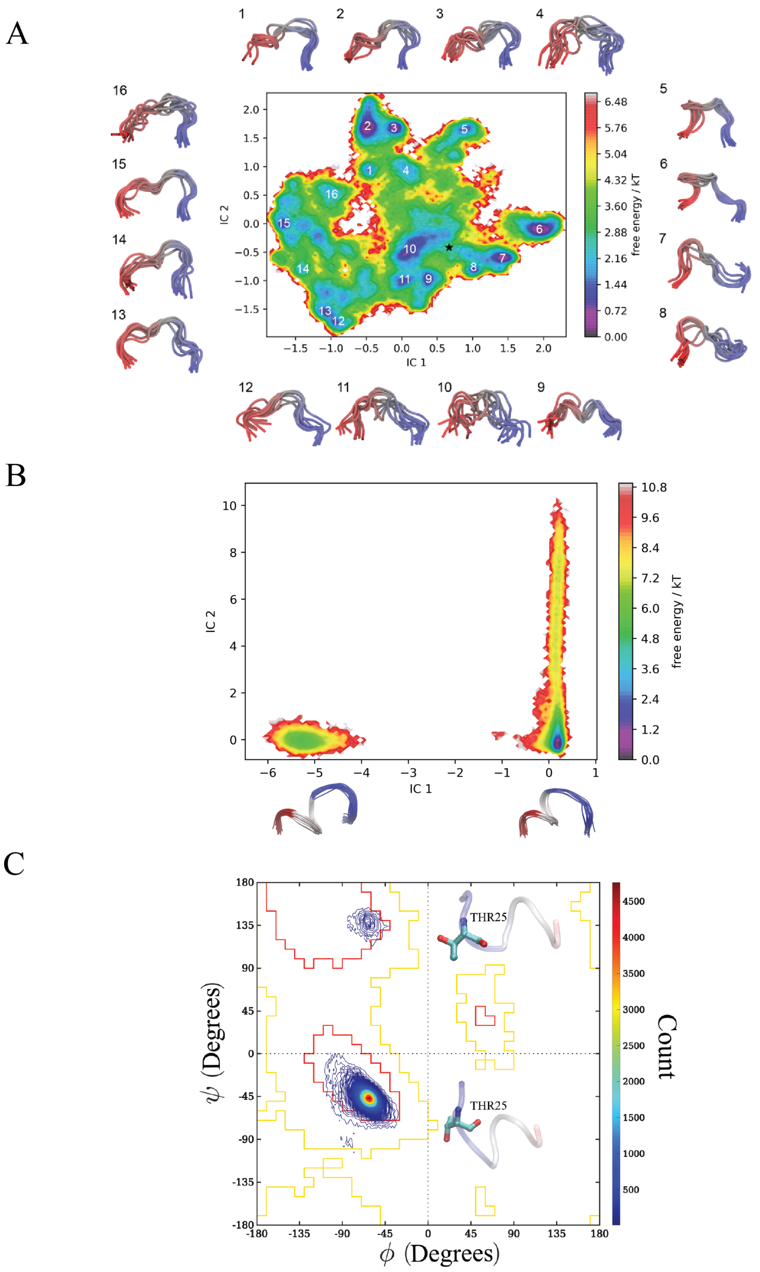

Supplementary Figure 8. Conformational dynamics of actin DNase-I binding loop (D-loop, residues 40 to 50) and α2-Helix (residues 23 to 31) derived by utilizing time-independent component analysis and equilibrium molecular dynamics simulations. The 1st and 2nd independent components (ICs) correspond to the major contributing components to the overall dynamics of the region of interest; independent components are like principal components but can capture nonlinear correlations. (A) Projection of the conformations observed for the D-loop trajectories onto the 1st and 2nd ICs, and its associated free energy profile. Projections of the D-loop trajectories reveal twelve free energy minima (labeled 2, 3, 5, 6, 7, 8, 9, 10, 11, 12, 13, and 15) and four saddle points (labeled 1, 4, 14, and 16); an ensemble of conformations is shown for each minimum or saddle point observed in the IC projections. The models are colored according to residue number, from residue 40 (red) to residue 50 (blue). (B) Projection of the α2-Helix trajectories onto the ICs space spanned by IC 1 and 2 and its associated free energy profile, reveals two major minima that are characterized by differences of the ψ dihedral torsion angle of THR25. The conformational ensemble corresponding to each minimum is shown; the models are colored from residue 23 (red) to residue 31 (blue). (C) Ramachandran plot of ϕ-ψ dihedral angles for THR25 reveals that the $\alpha2$-Helix samples two specific ψ dihedral angles with different probabilities.
