# Supplementary material for: Magic angle spinning NMR structure of human cofilin-2 assembled on actin filaments reveals isoform-specific conformation and binding mode
Source: Nat Commun. 2022 Apr 19;13:2114. doi: 10.1038/s41467-022-29595-9 (PMC9018683; doi:10.1038/s41467-022-29595-9)
Supplement: Supplementary file 2 — Reporting Summary [file 41467_2022_29595_MOESM2_ESM.pdf]

nature portfolio

Corresponding author(s): Tatyana Polenova

Last updated by author(s): February 17, 2022

Reporting Summary

Nature Portfolio wishes to improve the reproducibility of the work that we publish. This form provides structure for consistency and transparency in reporting. For further information on Nature Portfolio policies, see our Editorial Policies and the Editorial Policy Checklist.

Statistics

For all statistical analyses, confirm that the following items are present in the figure legend, table legend, main text, or Methods section.

n/a	Confirmed	
☐	☒	The exact sample size (*n*) for each experimental group/condition, given as a discrete number and unit of measurement
☐	☒	A statement on whether measurements were taken from distinct samples or whether the same sample was measured repeatedly
☒	☐	The statistical test(s) used AND whether they are one- or two-sided<br>*Only common tests should be described solely by name; describe more complex techniques in the Methods section.*
☐	☒	A description of all covariates tested
☒	☐	A description of any assumptions or corrections, such as tests of normality and adjustment for multiple comparisons
☒	☐	A full description of the statistical parameters including central tendency (e.g. means) or other basic estimates (e.g. regression coefficient) AND variation (e.g. standard deviation) or associated estimates of uncertainty (e.g. confidence intervals)
☐	☒	For null hypothesis testing, the test statistic (e.g. F, t, r) with confidence intervals, effect sizes, degrees of freedom and P value noted<br>*Give P values as exact values whenever suitable.*
☒	☐	For Bayesian analysis, information on the choice of priors and Markov chain Monte Carlo settings
☒	☐	For hierarchical and complex designs, identification of the appropriate level for tests and full reporting of outcomes
☒	☐	Estimates of effect sizes (e.g. Cohen's *d*, Pearson's *r*), indicating how they were calculated

Our web collection on statistics for biologists contains articles on many of the points above.

Software and code

Policy information about availability of computer code

Data collection	NMR data were collected using Bruker TopSpin version 3.5, a standard commercial program.
Data analysis	MAS NMR data were processed with Bruker TopSpin version 3.5 and NMRPipe version 8.7. NMR spectra were visualized and analyzed using CcpNmr Analysis version 2.4.2. Backbone dihedral angel restraints were generated from experimental NMR data using TALOS-N version 4.12. MAS NMR structures of CFL2 were calculated and refined in the Xplor-NIH 2.45.<br><br>Rigid body docking calculations of CFL2 into the cryo-EM density of cofilin (PDB ID: 3J0S) were performed using UCSF Chimera version 1.13.<br><br>Restraint tallying and format conversions were carried out with in-house Python 2.7 scripts. Structure ensembles were rendered for visualization in PyMOL 1.8.6.2 using in-house shell/bash scripts for batch rendering. Secondary structure elements were classified according to TALOS-N and manual inspection.<br><br>Molecular dynamics simulations were performed using NAMD version 2.14 and CHARMM36m protein and CHARMM TIP3P water force fields. Analysis of MD trajectories was performed using VMD.<br><br>Pyrene-actin fluorescence was recorded using i-Control 1.11 (Tecan Infinite M1000 Pro plate reader). TIRFM time-lapse images were collected using NIS Elements-AR (Nikon Eclipse Ti-E microscope). In vitro actin-based assays were analyzed using Microsoft Excel and open-source Fiji/ImageJ software.

For manuscripts utilizing custom algorithms or software that are central to the research but not yet described in published literature, software must be made available to editors and reviewers. We strongly encourage code deposition in a community repository (e.g. GitHub). See the Nature Portfolio guidelines for submitting code & software for further information.

Data

Policy information about availability of data

All manuscripts must include a data availability statement. This statement should provide the following information, where applicable:

- Accession codes, unique identifiers, or web links for publicly available datasets
- A description of any restrictions on data availability
- For clinical datasets or third party data, please ensure that the statement adheres to our policy

The coordinates corresponding to CFL2 have been deposited in the Protein Data Bank under accession code PDB 7M0G [http://doi.org/10.2210/pdb7M0G/pdb] for CFL2. MAS NMR chemical shift, distance restraints, and dihedral angle restraints have been deposited in the Biological Magnetic Resonance Data Bank (BMRB) under accession code 30877 [https://doi.org/10.13018/BMR30877]. The coordinates corresponding to actin filaments decorated with CFLGg used in this study are available in the Protein Data Bank under accession code PDB 5YU8 [http://doi.org/10.2210/pdb5YU8/pdb]. Additional coordinates corresponding to other proteins analyzed in this study are available in the Protein Data Bank under the following accession codes. The accession code for the solution NMR structure of CFLGg is PDB 1TVJ [http://doi.org/10.2210/pdb1TVJ/pdb] and corresponding BMRB entry 5177 [https://doi.org/10.13018/BMR5177]. The accession code for actin filaments decorated with CFL1 is PDB 6VAO [http://doi.org/10.2210/pdb6VAO/pdb].

Field-specific reporting

Please select the one below that is the best fit for your research. If you are not sure, read the appropriate sections before making your selection.

- [x] Life sciences
- [ ] Behavioural & social sciences
- [ ] Ecological, evolutionary & environmental sciences

For a reference copy of the document with all sections, see nature.com/documents/nr-reporting-summary-flat.pdf

Life sciences study design

All studies must disclose on these points even when the disclosure is negative.

Sample size	All NMR samples are described in the methods. The sample sizes are standard for the in vitro assays performed in the study.
Data exclusions	No data were excluded from the analyses.
Replication	Multiple samples have been measured by solid-state NMR, as described in the text, with consistent results. The molecular assays performed in this study were replicated two or three times. All replications were successful.
Randomization	Randomization was not relevant to the molecular assays performed in this study, as no human or animal subjects were studied, and the collected physical data were quantitative and did not require subjective interpretations.
Blinding	Blinding was not relevant to this study, as no subjective allocation was involved, and the results were quantitative and analyzed without subjective manual scoring.

Reporting for specific materials, systems and methods

We require information from authors about some types of materials, experimental systems and methods used in many studies. Here, indicate whether each material, system or method listed is relevant to your study. If you are not sure if a list item applies to your research, read the appropriate section before selecting a response.

Materials & experimental systems

n/a	Involved in the study	
x		Antibodies
x		Eukaryotic cell lines
x		Palaeontology and archaeology
x		Animals and other organisms
x		Human research participants
x		Clinical data
x		Dual use research of concern

Methods

n/a	Involved in the study	
x		ChIP-seq
x		Flow cytometry
x		MRI-based neuroimaging
